# Formation and growth mechanism for niobium oxide nanoparticles: atomistic insight from *in situ* X-ray total scattering†

**DOI:** 10.1039/d0nr08299f

**Published:** 2021-03-15

**Authors:** Olivia Aalling-Frederiksen, Mikkel Juelsholt, Andy S. Anker, Kirsten M. Ø. Jensen

**Affiliations:** Department of Chemistry and Nano-Science Center, University of Copenhagen 2100 Copenhagen Ø Denmark kirsten@chem.ku.dk

## Abstract

Understanding the mechanisms for nanoparticle nucleation and growth is crucial for the development of tailormade nanomaterials. Here, we use X-ray total scattering and Pair Distribution Function analysis to follow the formation and growth of niobium oxide nanoparticles. We study the solvothermal synthesis from niobium chloride in benzyl alcohol, and through investigations of the influence of reaction temperature, a formation pathway can be suggested. Upon dissolution of niobium chloride in benzyl alcohol, octahedral [NbCl_6−*x*_O_*x*_] complexes form through exchange of chloride ligands. Heating of the solution results in polymerization, where larger clusters built from multiple edge-sharing [NbCl_6−*x*_O_*x*_] octahedra assemble. This leads to the formation of a nucleation cluster with the ReO_3_ type structure, which grows to form nanoparticles of the Wadsley–Roth type H-Nb_2_O_5_ structure, which in the bulk phase usually only forms at high temperature. Upon further growth, structural defects appear, and the presence of shear-planes in the structure appears highly dependent on nanoparticle size.

## Abstract 2

Understanding the mechanisms for nanoparticle nucleation and growth is crucial for the development of tailormade nanomaterials.

## Graphical abstract

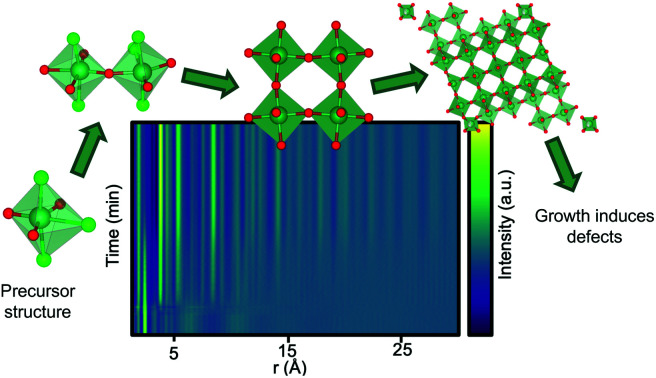

## Introduction

Niobium oxides have in recent years received significant interest due to their versatile electrochemical properties. For example, niobium and niobium-based oxide materials have been shown to be outstanding cathode materials for high-power batteries due to high Li-diffusion rates through their crystal structures.^1–7^ Additionally, studies have shown that niobium oxides show excellent potential as catalyst materials for production of *e.g.* fuels and chemicals from biomass sources.^8–10^ Niobium oxides have furthermore been extensively studied for their electrochromic properties *e.g.* photodetection^11^ and in recent years, the potential of amorphous and nanostructured Nb_2_O_5_ for electrochromic windows has been investigated.^12–14^

**Fig. 1:**
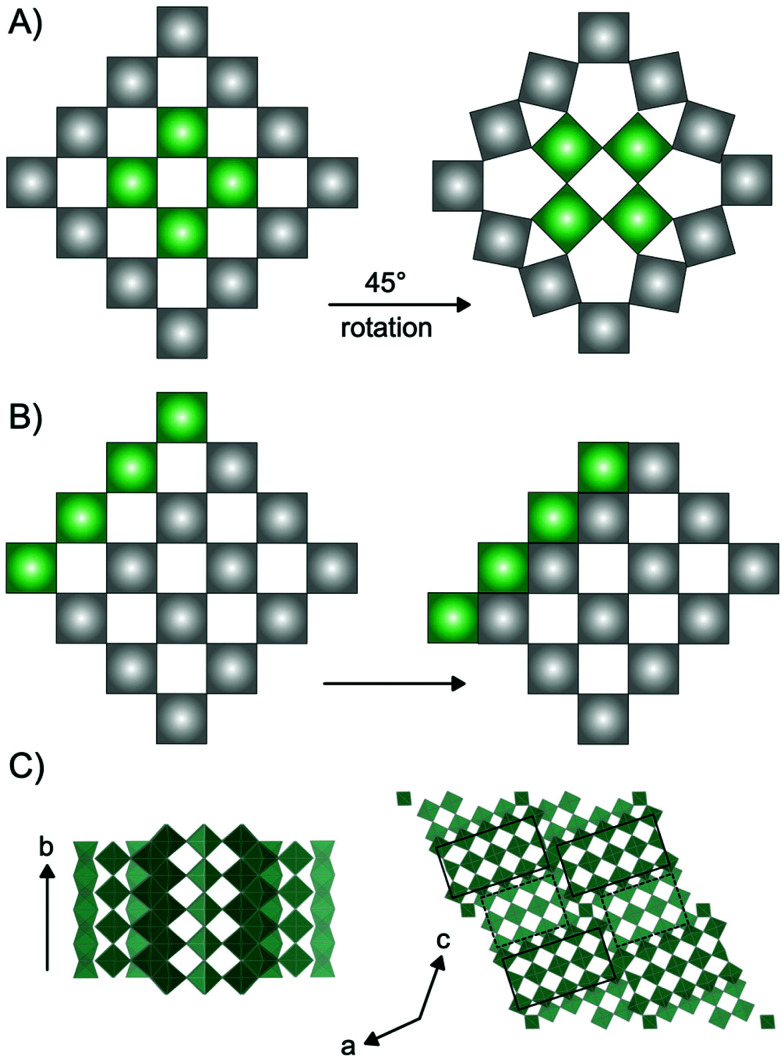
Illustrations of the formation of extended defects in ReO_3_ structures. Each square represents an octahedron with a metal atom in the center and oxygen atoms in the corners. (A) Tetragonal Tungsten Bronze-type (TTB) structure forming from a 45° rotation of the highlighted green octahedra in the ReO_3_-type structure. (B) Formation of shear planes from a movement of octahedra illustrated by the highlighted green squares. Shear planes are present in both Magnéli phases and Wadsley–Roth type structures. (C) H-Nb_2_O_5_ along different axes. The black squares represent blocks of (3 × 5) and (3 × 4) ReO_3_ type octahedra within the structure.

The versatility of applications of niobium-based oxides arises from their rich structural chemistry.^9,15^ The building block of niobium pentoxide Nb_2_O_5_ is the [NbO_6_] octahedra which occur in the oxide structures as corner-sharing or edge-sharing, *i.e.* sharing one or two oxygen atoms, respectively. The many opportunities in the arrangement of these octahedra allow for the structural diversity, and a range of Nb_2_O_5_ polymorphs as well as non-stoichiometric phases exist. Many of these are based on the ReO_3_ structure, which is a cubic crystal structure in space group *Pm*3̄*m*, built from only corner-sharing [ReO_6_] octahedra. Extended defects in the ReO_3_ structure can lead to a range of phases with distinct structural differences. Such extended defects are commonly seen in early transition metal oxides such as niobium oxides, as well as vanadium oxides,^16^ molybdenum oxides^17^ and tungsten oxides.^18^ As illustrated in Fig. 1, they appear in different ways: as tetragonal tungsten bronzes (TTB-type defects),^19^ as Magnéli phases^18^ and as block-type structured Wadsley–Roth phases.^20^ The TTB-type structure (Fig. 1A) is found in *e.g.* mixed niobium oxides (Nb_2_WO_8_) and tungsten oxides (W_32_O_82_), as well as in the T-Nb_2_O_5_, and the related TT-Nb_2_O_5_ structure. The structure originates from cubic ReO_3_, however, a 45° rotation of four octahedra in the *ab*-plane leads to pentagonal columns in the structure.^19^ The transformation from the parent to the TTB-type structure furthermore results in a distortion of the octahedra, and the interstitial site formed in the pentagonal columns can be either partially or fully occupied by metal atoms.^21^ Magnéli phases (Fig. 1B) also originate from the ReO_3_-type structure but contain slabs of edge-sharing octahedra in two directions parallel to one of the unit cell axes. This leads to ReO_3_ domains which are infinite in two directions, but finite in the third. The formation of such shear planes has been explained by supersaturation of oxygen vacancies resulting in a collapse of the lattice.^22^ Magnéli phases are observed in particular in molybdenum-, vanadium- and titanium oxides. In niobium oxides, however, Wadsley–Roth block-type structures are more commonly seen. The block-type structures (illustrated in H-Nb_2_O_5_ in Fig. 1C) have finite blocks of the ReO_3_ structure in a two-direction perpendicular plane, separated by shear planes along (100) and (001). Many Nb_2_O_5_ phases and nonstoichiometric niobium oxide phases (*e.g.* H-Nb_2_O_5_, M-Nb_2_O_5_, N-Nb_2_O_5_, Nb_12_O_29_ and Nb_22_O_54_) have this block-type structure. The block size, meaning the number of corner-sharing octahedra within a block, differ between the phases, and various sizes of blocks can be present in one phase.^20^ An effect of crystallographic shear planes in the block structure of ReO_3_ is that metal atoms in tetrahedral coordination often appears in the intersection between blocks, as is observed in the H-Nb_2_O_5_ in Fig. 1C. The monoclinic H-Nb_2_O_5_ structure is reported to be the most thermodynamically stable of the niobium pentoxides^15^ and it is usually formed at high temperature (>900 °C). It contains blocks of two different sizes, (3 × 4) octahedra and (3 × 5) octahedra separated by crystallographic shear planes.^15,23^

In Table S1† we give an overview of the most important reported niobium oxides and their crystal structure. We note here that the nomenclature of the different Nb_2_O_5_ phases can easily lead to a lot of confusion. Where H-Nb_2_O_5_, M-Nb_2_O_5_, T/TT-Nb_2_O_5_ are named from the temperature at which they are obtained (German: Hoch, Medium, Tief), B-Nb_2_O_5_, N-Nb_2_O_5_ and P-Nb_2_O_5_ have their names from crystallite shapes (German: Blätter, Nadeln, Prismen).^15,24^ While niobium oxides have been studied for decades, the complex defect chemistry furthermore means that the structures of many of these phases are still debated, and much uncertainty exists on the number of niobium oxide polymorphs and their crystal structures.

The large structural diversity in niobium oxides, along with the strong structure–property relation, means that structure control during synthesis is crucial in material preparation, and much work has been devoted to the synthesis of niobium oxide nanomaterials. While heat treatment of NbO_2_ in solid state reactions is the simplest way to obtain different modifications of bulk, crystalline Nb_2_O_5_,^3,24^ hydro- and solvothermal synthesis have in general proven to be efficient methods for nanomaterial preparation.^25,26^ Several studies have demonstrated the synthesis of niobium based oxide nanosheets,^1,27^ nanorods^7,28^ and nanospheres^8^ through hydro- and solvothermal synthesis. Other approaches, such as colloidal synthesis methods^14^ and electrospinning^4^ have been reported. However, very little is known on the atomic-scale reaction mechanisms that take place during material synthesis, which hinders rational design of synthesis methods for a given niobium oxide structure.

*In situ* X-ray total scattering studies is an effective way of following chemical reactions as they take place, revealing important information on the formation mechanism of *e.g.* various metal oxides.^29–35^ The use of Pair Distribution Function (PDF) analysis of total scattering data means that structural information can be obtained for matter with and without long-range order, making it possible to follow the structural rearrangements that take place in a synthesis all the way from ionic or molecular clusters in solution to the final material.^36,37^ Here, we study the solvothermal synthesis of Nb_2_O_5_ nanoparticles from NbCl_5_ in benzyl alcohol, first described by Buha *et al.*^38^ This non-aqueous sol–gel route, applying a metal chloride precursor in benzyl alcohol, can be used for synthesis of a range of different oxide materials, also for compounds that are otherwise difficult to prepare, such as niobium oxides.^39,40^ The solvothermal synthesis approach is furthermore simple and industrially relevant, as it can easily be scaled up. Benzyl alcohol has proven an efficient solvent for control of particle size, phase and crystallinity^40^ and acts as oxygen supplier, capping agent and solvation medium.^41^ Deshmukh *et al.*^39^ have suggested a reaction scheme for the reaction between metal halides and benzyl alcohol, where a metal hydroxide species first form as an intermediate structure through hydrolysis. However, further details on the possible hydroxide species are unknown, as the reaction rapidly takes place even at room temperature.^39^ For the niobium oxide synthesis in particular, the mechanisms leading to a specific atomic structure is unknown. From our *in situ* data, we can suggest a formation mechanism for niobium oxide nanoparticles, where single [NbO_6_] octahedral building blocks first assemble to form a nucleation cluster of a ReO_3_ type network. This structure further grows to form the final structure: a Wadsley–Roth block-structure phase. Formation of [NbO_6_] octahedra takes place through exchange of chloride ligands, and assembly of the octahedra happens as soon as the chloride ligands have been exchanged. We furthermore show how synthesis temperature and applied pressure influence the final structure and induce the formation of shear planes and block structures in the material. Such knowledge gives a new approach to structure control of niobium oxides by synthesis design.

## Experimental methods

Niobium oxide synthesis was carried out using a solution of NbCl_5_ (99%, Sigma Aldrich) in benzyl alcohol (Assay (GC, area%): ≥99.0% (a/a), Sigma Aldrich).^38^ The synthesis was done in our custom-made reaction cell for *in situ* X-ray studies of solvothermal synthesis, which is similar in design to that previously described by Becker *et al.*^42^ The precursor suspension was injected into a fused silica tube with 0.7 mm inner diameter and 0.09 mm wall thickness. A HPLC pump was used to pressurize the tube and a hot air blower was used to reach the desired temperature within the tube. Four different conditions were studied, 160 °C, 200 °C and 300 °C at 100 bar pressure, and 100 °C at ambient pressure. A high concentration of NbCl_5_ in benzyl alcohol is necessary to obtain enough scattering signal for PDF analysis, and the precursor was prepared to yield 0.6 M NbCl_5_. However, the exact concentration in the reaction cell is associated with some uncertainty due to the lack of complete solubility of the NbCl_5_ in benzyl alcohol.

The *in situ* X-ray total scattering experiments were performed at P02.1 PETRA III, DESY, Germany with a wavelength of *λ* = 0.2072 Å. The RA-PDF^43^ geometry was applied using a large 2D detector, measuring 40 by 40 cm and a 216.9 mm sample to detector distance. The time resolution of the collected data was 5 s. The collected 2D data were integrated using Fit2D,^44^ and the total scattering data were Fourier Transformed to obtain PDFs using PDFgetX3.^45^ The following parameters were used for the data reduction: *Q*_min_ = 0.6 Å^−1^, *Q*_max_ = 14.5 Å^−1^, *Q*_maxinst_ = 22.4 Å^−1^ and rpoly = 0.9 Å. Before the Fourier transformation, the background scattering signal from the fused silica capillary and the pure solvent at the appropriate temperature and pressure was subtracted. The background subtraction is described in detail in the ESI, Fig. S2.†

The PDFs obtained from crystalline nanoparticles were modelled using PDFgui,^46^ where real-space Rietveld refinements were performed. Cluster structures with no long-range order were modelled using Diffpy-CMI^47^ with the DebyePDFCalculator. The modelling is described in detail below and in the ESI.†

Niobium oxide NPs were also synthesized for *ex situ* characterization. Here, a 3 mm borosilicate NMR tube was filled with the 0.6 M NbCl_5_ solution, sealed with a septa and lowered into a 100 °C hot oil bath. After 13 minutes of heating, the substance was transferred into a borosilicate glass capillary with an outer diameter of 0.8 mm for X-ray total scattering data collection. Total scattering data were collected at room temperature using a Panalytical Empyrean Series 2 diffractometer with an Ag-source (X-ray wavelength of 0.56 Å), and a GaliPIX detector. An identical measurement was performed with a borosilicate glass capillary with pure benzyl alcohol and used in the background subtraction. The as-prepared suspension was furthermore washed with ethanol, and a dilute suspension was prepared and drop casted on a lacey carbon grid and transmission electron microscopy (TEM) images were collected on a Tecnai T20 G2 200 kV TEM. The images were further processed with the FIJI software.^48^

Small Angle X-ray Scattering (SAXS) data were collected from the as-prepared suspension. The sample was loaded into a quartz capillary with outer diameter of 1.0 mm, and SAXS data were measured using a SAXSLab instrument (JJ-XRay) with a Rigaku 100 XL+ micro focus sealed X-ray tube and a Dectris 2D 300 K Pilatus detector with X-ray wavelength of 1.54 Å. Data from benzyl alcohol and an empty quartz capillary were measured as backgrounds. The data were integrated using the programme Saxsgui and simulated SAXS formfactor models were calculated with Diffpy-CMI, which uses SasView^49^ functions.

## Results and discussion

### Formation of Wadsley–Roth structured Nb_2_O_5_

**Fig. 2:**
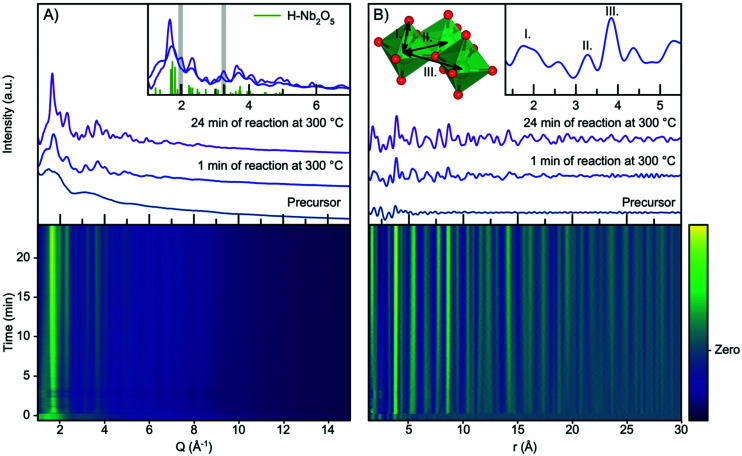
(A) Time resolved XRD data obtained during the formation of niobium oxide nanoparticles at 300 °C. Selected patterns are plotted, representing the precursor, the product after 1 min, and the product obtained after 24 min. A calculated pattern from the H-Nb_2_O_2_ structure is included in the inset and two peaks at 2.0 Å^−1^ and 3.3 Å^−1^are highlighted. (B) Time resolved PDF with selected PDFs plotted as in (A). The insets show a cutout from the Nb_2_O_5_ crystal structure with assignment to PDF peaks.

Fig. 2A shows the time resolved scattering data obtained during niobium oxide synthesis at 300 °C. The data are shown as a contour plot, along with three selected scattering patterns from different stages of the reaction (precursor, 1 minute of reaction and 24 minutes of reaction). The synthesis is initiated by applying heating at *t* = 0 minutes, and the temperature reaches 90% of the setpoint temperature after the first 10 seconds. A rapid change in the scattering pattern is observed as the heat is turned on, and a nanocrystalline phase forms almost instantly as seen from the emergence of broad Bragg peaks. As the reaction progresses, the peaks become more pronounced and sharper as seen when comparing the data obtained at 1 minute and 24 minutes into the reaction.

The observed scattering patterns are compared with calculated Bragg peak intensities of H-Nb_2_O_5_ in Fig. 2A, inset. A comparison with diffraction patterns calculated from other niobium oxides is shown in Fig. S4.† None of the known niobium oxide crystal structures fully describe the observed scattering patterns, although H-Nb_2_O_5_ accounts for the main features in the pattern, especially for the particles formed at the beginning of the reaction. Other features at *Q*-values 2.0 Å^−1^ and 3.3 Å^−1^, highlighted in Fig. 2A inset, evolve during the reaction. We have previously observed that the presence of extended defects in nanomaterials is highly size-dependent,^50^ and it is likely that the particle growth during this reaction induces defects in the structure. However, the large Bragg peak broadening from nanoparticle size makes a conventional crystallographic analysis of these effects difficult, and we therefore turn our investigation to the Pair Distribution Function (PDF) of the total scattering data.

The time resolved PDFs obtained from the total scattering data are shown in Fig. 2B. As expected from the *Q*-space data, it is evident from the PDFs that the nanocrystalline phase form immediately as the reaction is initiated as the PDF oscillations extend to higher *r*, whereas the PDF collected for the precursor shows limited structural coherence. We discuss the structure of the precursor, as well as the nucleation mechanism, in a section below, and first investigate the PDF describing the structure of the nanoparticles forming.

Fig. S5† shows a comparison between the experimental PDF obtained after 1 minute and PDFs calculated from known niobium oxide crystal structures. As expected, the plot shows that many of the structures are very similar locally as they share a structural motif of corner-sharing and edge-sharing [NbO_6_] octahedra. As shown in the inset in Fig. 2B, the PDF peaks at 3.3 Å and 3.8 Å arise from Nb–Nb pairs in edge- and corner-sharing geometry,^51^ respectively, while the broad peak at 1.8 Å arises from Nb–O distances within the octahedra.^52^ By considering the intensity ratio between the edge- and corner-sharing peaks, it is clear that the product from the synthesis is closer in structure to the Wadsley–Roth structures than the TTB-structured T-Nb_2_O_5_, which does not give a good description of our data. This is surprising, as the product from a very similar synthesis was found by Buha *et al.*^38^ to take the TT-Nb_2_O_5_ structure. The TT-phase is expected to be similar in structure to the T-phase, although its structure has never really been resolved.^53^ We expect the deviation from earlier studies observed here to be an effect of the experimental conditions, *e.g.* the higher heating rate in the current experiment compared with a conventional solvothermal synthesis using autoclaves, the difference in pressure within the reaction vessels, and the reaction container material. The formation of the H-Nb_2_O_5_ phase at the low reaction temperature used here is notable, as this phase usually forms at much higher temperature, (>900 °C), at least in the case of bulk materials.^15^

**Fig. 3:**
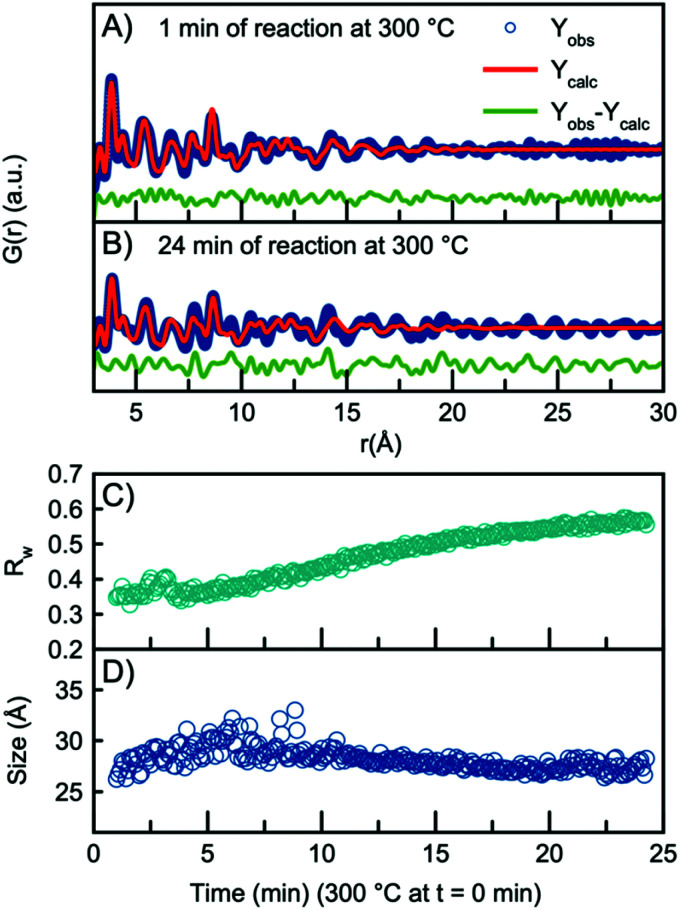
PDF fit using the H-Nb_2_O_5_ structure to the data collected (A) after 1 min of reaction and (B) 24 min of reaction. (C) *R*_w_ values obtained from sequential refinement with H-Nb_2_O_5_ plotted as a function of time. (D) The refined crystallite size plotted as a function of time.

As the H-Nb_2_O_5_ has been reported as the most thermodynamically stable of the Wadsley–Roth structured Nb_2_O_5_ polymorphs,^15^ we use this structure as a starting model for further structural analysis. A fit to the data obtained after 1 minute at 300 °C using the H-Nb_2_O_5_ model is plotted in Fig. 3A, showing good agreement between model and data. A description of the refinement strategy and the refined parameters can be found in Table S2.† Note that the fits shown only include the *r*-range from 3–30 Å, thus not including the first peak originating from the first Nb–O distance, which can be seen in Fig. 2B to be highly asymmetric. The evolution of the Nb–O peak will be discussed further below.

The refined crystallite size from the fit in Fig. 3A is 26 Å, however, the fit clearly shows weak features at higher *r*-values which are not well described by the model. In fact, the refined crystallite size is slightly smaller than a single H-Nb_2_O_5_ unit cell in the *a*/*c* plane, 35 Å. After 24 minutes of reaction, the misfit at higher *r*-values becomes much more pronounced as seen in the fit in Fig. 3B. The increased misfit with time is clearly observed in Fig. 3C, where the fit *R*_w_-values are plotted, representing the decreasing quality of the fit when performing sequential refinements. The evolution of the refined crystallite size (sp-diameter, Fig. 3D) in the sequential refinements furthermore indicates that the H-Nb_2_O_5_ model cannot describe the long-range order of the PDF as it cuts off the crystallite size at ∼30 Å even though the features in the experimental PDF extend to ∼45 Å (Fig. S6†). Sequential refinements with other Wadsley–Roth niobium oxide phases are shown in Fig. S8,† giving very similar results, and all reported phases give a poor description of the long-range order observed in the PDF obtained at the end of the experiment.

**Fig. 4:**
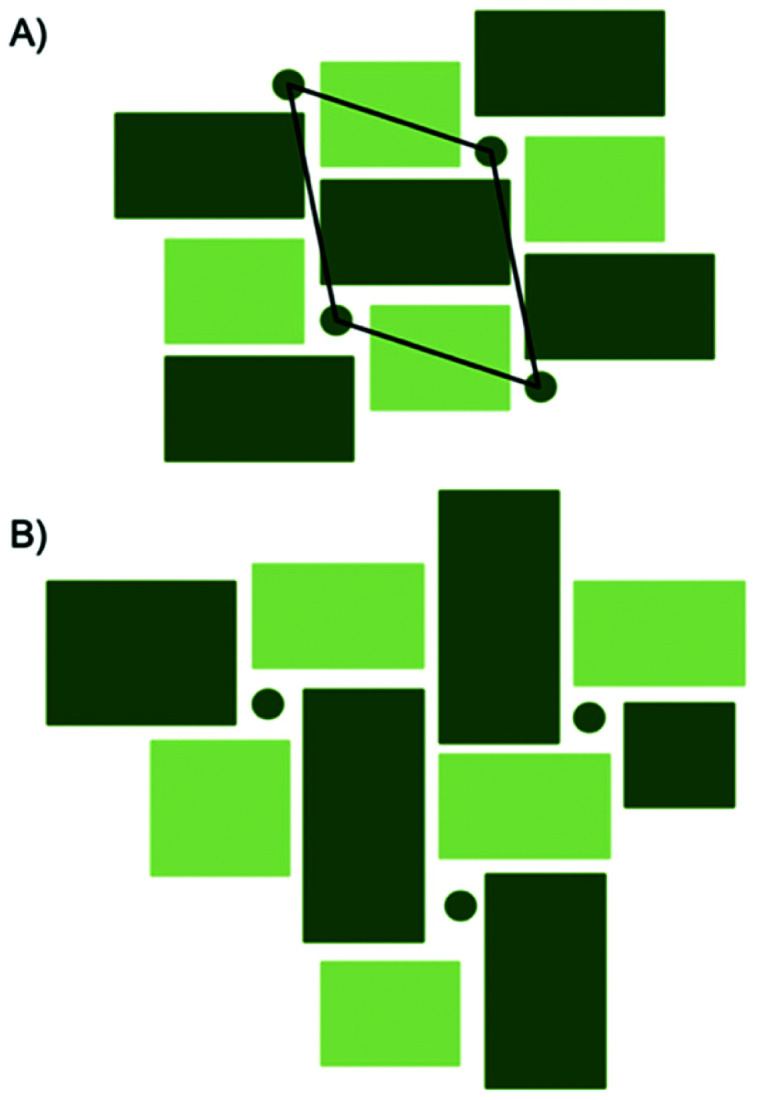
(A) Idealized representation of H-Nb_2_O_5_. Dark green circles represent tetrahedral sites and the dark and light green squares represent ReO_3_ type blocks. The black rhombus illustrates the unit cell of the H-Nb_2_O_5_. (B) A distorted version of H-Nb_2_O_5_ with several different block sizes and no systematic ordering.

A comparison between the PDFs obtained at 1 minute and 24 minutes in Fig. 2B and S6† show that with time, the peak arising from edge-sharing [NbO_6_] octahedra becomes more prominent. The H-Nb_2_O_5_ structure (Fig. 1C) is built up from ReO_3_ blocks of (3 × 5) and (3 × 4) corner-sharing octahedra, where edge-sharing occur only between blocks. This is further illustrated in Fig. 4A. The block size in Nb_12_O_29_ are (3 × 4) and in Nb_22_O_54_ (3 × 4) and (3 × 3) octahedra. The good agreement between data and model in the low *r*-range when fitting with these three structures, combined with the large misfit at high *r*-values, indicate that the particles formed in the solvothermal synthesis are indeed built up from ReO_3_ block structures, but that the block sizes and arrangement may differ from the well-ordered, known and well-characterized bulk structures. HR-TEM analysis has previously shown that disordered regions can be present in niobium oxide nanoparticles. For example, in 1973 IIjima showed the presence of blocks of different sizes assigned as heavily disordered regions in the H-Nb_2_O_5_,^54^ and several studies conducted in the 1970s show similar imperfections in the block structure of Nb_2_O_5_.^55–57^ More recently, Russo *et al.*^8^ showed that T-Nb_2_O_5_ nanoparticles synthesized through solvothermal synthesis with NbCl_5_ and acetophenone have regions deviating from the crystalline nature of the material. Fig. 4 shows an illustration of these block defects found in niobium oxide particles compared to the idealized H-Nb_2_O_5_ structure. While the idealized H-Nb_2_O_5_ in Fig. 4A has two distinctive block sizes illustrated by dark and light green squares, the defect structure shown in Fig. 4B contains various sizes of blocks with no systematic ordering. In a recent study of molybdenum oxide nanoparticles, we saw that nanosizing in a similar way can lead to disordered shear-planes rather than the well-ordered Magneli phases from bulk materials.^50^

**Fig. 5:**
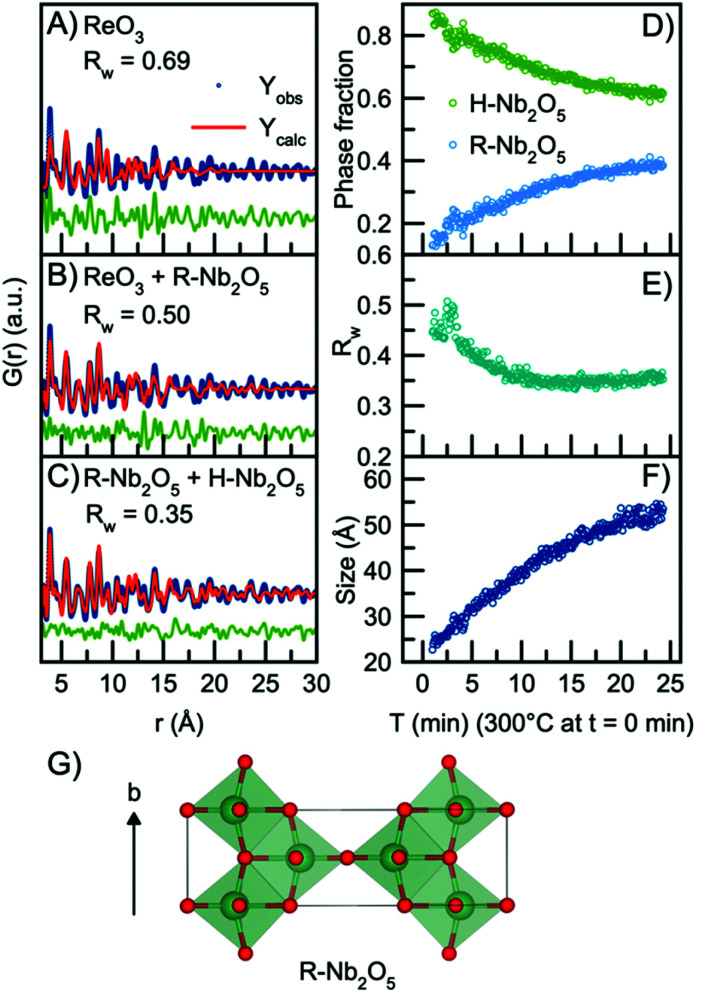
Real-space Rietveld refinement of the PDF collected 24 min into the reaction using the structural model (A) ReO_3_, (B) ReO_3_ and R-Nb_2_O_5_, (C) R-Nb_2_O_5_ and H-Nb_2_O_5_. (D) Refined phase fractions of H-Nb_2_O_5_ and R-Nb_2_O_5_ plotted as a function of time. (E) Fit agreement factor *R*_w_ plotted as a function of time. (F) The refined crystallite size (sp-diameter) plotted as a function of time. The crystallite size for the H- and R-phases were constrained to take the same value. (G) The crystal structure of R-Nb_2_O_5_ first reported by Gruehn.^58^ The structure is built from corner-sharing and edge-sharing octahedra.

In order to model the long-range order of the particles, we therefore again look towards different Nb_2_O_5_ crystal structures. The R-Nb_2_O_5_ phase shown in Fig. 5G was first reported by Gruehn in 1966.^58^ Here, a range of different synthesis methods were shown to result in R-Nb_2_O_5_ along with other phases like TT- and a P-Nb_2_O_5_ phase, which was reported to be highly similar in structure compared with the R-Nb_2_O_5_. The structures and existence of these phases are however associated with much uncertainty, and to the best of our knowledge, the crystal structure of P-Nb_2_O_5_ has not been reported. Since the first synthesis of R-Nb_2_O_5_ in 1966, the P/R-Nb_2_O_5_ phases have rarely occurred in the literature and have mostly been described in computational studies of niobium oxides. For example, Valencia-Balvin *et al.*^59^ performed a computational study where they showed that R-Nb_2_O_5_ is metastable compared to other structural modifications. Therefore, the existence of these modifications is doubtful and more thorough clarification of the structural chemistry of niobium pentoxide is still needed. However, R-Nb_2_O_5_ is the simplest of the niobium pentoxide modifications. Interestingly, its structure is similar to the shear planes forming between block structures in Wadsley–Roth phases as seen in Fig. 1B and C. Fig. 5B shows a fit to the data obtained after 24 minutes of reaction using this R-Nb_2_O_5_ structure along with the simple ReO_3_ structure, while a fit using only the ReO_3_ structure is shown in Fig. 5A. The refinement values are presented in Tables S7 and 8.† These fits illustrate that many features from the local structure can be described by the ReO_3_ motifs, while peaks at higher *r*-values are included by the model from R-Nb_2_O_5_. A better result is obtained when combining the R-Nb_2_O_5_ structure with H-Nb_2_O_5_ as seen in Fig. 5C.

Compared to the single-phase fit with H-Nb_2_O_5_ in Fig. 3B, the agreement factor *R*_w_ decreases from 0.55 to 0.35 when including the R-Nb_2_O_5_ phase. The model clearly does not fully describe all features of the PDF intensities; however, all main PDF peaks are now included. We do not interpret this as the occurrence of a secondary phase during Nb_2_O_5_ synthesis, but rather that the combination of the H- and R-structure accounts for the development of Wadsley–Roth structures with blocks of different sizes, separated by a R-Nb_2_O_5_-like interface. In the model, the H-structure thus mainly describes the structure of the blocks, while the R-structure may account for the interface between them. The existing misfit in the PDF when modelling with two different phases likely arises from the heterogeneous nature of the particles, where blocks of different sizes may be present as illustrated in Fig. 4B. This effect is not included in the simple model applied here.

Through sequential refinement with the two-phase model with both H- and R-Nb_2_O_5_, we can follow the structural changes after initiation of the reaction. Model details and representative fits from different time points during the reaction is given in the ESI, Fig. S9 and Table S9.†Fig. 5D shows the weight phase fraction of the H- and R-Nb_2_O_5_ phase plotted as a function of time. While the phase fraction of H-Nb_2_O_5_ decreases, the phase fraction of R-Nb_2_O_5_ increases. We interpret this as the formation of disordered block structures, and as the occurrence of increased [NbO_6_] edge-sharing in the structure.

Fig. 5E illustrates that the *R*_w_ values stay between 0.35 and 0.45 for all time points in the reaction, showing that the model gives an adequate description of the data for all reaction stages, unlike the fit with the single H-Nb_2_O_5_ phase. The crystallite size for the two phases was constrained to take the same value, and the particle growth is illustrated in Fig. 5F, with the crystallite size growing from *ca.* 2.5 nm to 5 nm, as expected from the evolution of the PDFs. No significant changes in the refined unit cell parameters for the two structural models are observed in the sequential refinement of the reaction process which indicates a stable refinement, Fig. S10 and 11.† The refinements show an increase in the oxygen atomic displacement parameters, which is an additional indication of increasing disorder in the structure with time and particle growth.

As noted above, we have not included the first Nb–O pair in the refinement, which is the first peak in the PDF. In the contour plot in Fig. 2B (and more clearly in Fig. S7†), we see that with synthesis time, this peak broadens and splits into two, centered at *ca.* 1.7 Å and 2.1 Å. The splitting is expected to be related to the formation of the defect structure, with different domains and structural motifs present. The shorter Nb–O distance at 1.7 Å agrees with what has previously been reported for the R-Nb_2_O_5_ phase.^58^

**Fig. 6:**
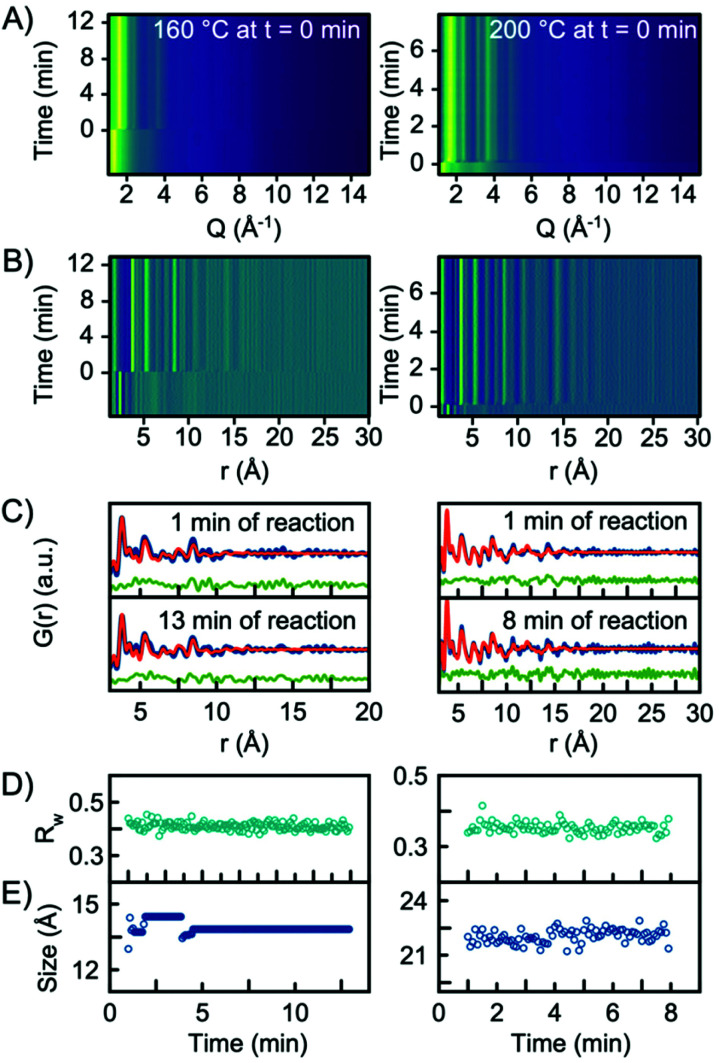
(A) Time resolved XRD data and (B) PDFs obtained from experiments conducted at 160 °C and 200 °C. (C) PDF fit of data collected 1 min into the reaction, and at the end of the experiments using H-Nb_2_O_5_ as the structure model. (D) *R*_w_ values obtained from sequential refinements with the H-Nb_2_O_5_ model plotted as a function of time. (E) The refined crystallite size (sp-diameter) plotted as a function of time.

*In situ* experiments were also carried out at reaction temperatures of 160 °C and 200 °C. Fig. 6A and B show the *Q*-space data along with the PDFs obtained for the two experiments. When fitting the data with a single-phase H-Nb_2_O_5_ structure, the model gives an adequate description of the PDF, and it is not necessary to include the R-Nb_2_O_5_ structure in the model, Fig. 6C. In fact, implementing a second phase of R-Nb_2_O_5_ in a two-phase refinement of the 160 °C and 200 °C data resulted in a negative refined value of the R-Nb_2_O_5_ scale factor. The refinement values using the H-Nb_2_O_5_ structural model are shown in Tables S3 and 4.† The refinements showed a crystallite size of ∼1.5 nm and ∼2 nm for the 160 °C and 200 °C experiments respectively, compared to ∼5 nm for 300 °C. As shown in Fig. 6E, we observe no growth of the particles upon heating as was the case for the experiment performed at 300 °C. We furthermore observe no change in the scale factor for the two experiments, as illustrated in Fig. S12 in the ESI.† Combined, the evolution of the scale factors and the growth curves indicate that all precursor material nucleates into the nanocrystalline phase as the heat is turned on. No further material nucleation or particle growth happens when the synthesis temperature is 200 °C or below, within the experimental time investigated here. The fact that the structure of the smaller particles synthesized at 160 °C and 200 °C is well-described by the H-Nb_2_O_5_ structure again emphasizes that the defect chemistry of the particles formed in the solvothermal synthesis is highly size-dependent.

### Analysis of precursor structure

**Fig. 7:**
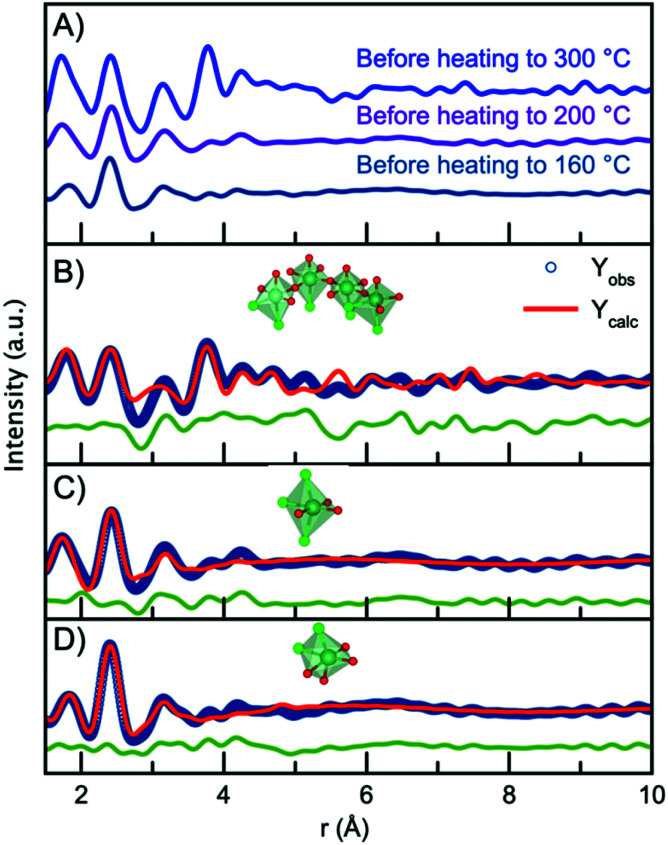
(A) PDFs collected for the precursor for each of the three experiments. (B) PDF fit to the data obtained for the 300 °C precursor, using a chain of [NbCl_6−*x*_O_*x*_] octahedra. (C) PDF fit to the data obtained for the 200 °C precursor, using a single [NbCl_3_O_3_] octahedra. (D) PDF fit to the data obtained for the 160 °C precursor, using a single [NbCl_3_O_3_] octahedra.

Having established the structure of the end product of the synthesis, we can now consider the structure present before nucleation of the nanocrystalline phase in order to deduce a nucleation mechanism. Fig. 7A shows the PDFs obtained from the precursors for the nanoparticles synthesized at the three different temperatures (300 °C, 200 °C and 160 °C). The data are obtained at room temperature, *i.e.* before the experiments are initiated. Surprisingly, the three PDFs show different features, which are most likely due to ageing of the precursor solution, as will be discussed below. First, we analyze the precursor PDF obtained from the 300 °C experiment. This PDF shows several sharp peaks, which can be assigned to interatomic distances by considering the chemistry of the NbCl_5_ precursor. In this structure, Nb^5+^ is octahedrally coordinated to Cl^−^ with bond distances ranging from 2.2 Å to 2.6 Å,^60^ agreeing with the sharp peak present at 2.4 Å. However, we also notice a peak at 1.8 Å agreeing with a Nb–O distance. This suggests that upon dissolution of NbCl_5_ in benzyl alcohol there is both a breakdown of the NbCl_5_ crystal structure, as well as a substitution of chloride for oxygen, possibly as a hydroxide or alkoxide group. The presence of PDF peaks at higher *r*-values also illustrates the formation of a structure beyond individual octahedra. To assign these peaks to interatomic distances, we look towards niobium oxychloride structures, such as crystalline Cs_2_[Nb_3_O_5_Cl_7_] which is a layered-type structure built from [CsCl_10_] layers and [NbOCl] layers,^61^ shown in Fig. S13.† By considering the structural motifs in this phase, we can assign the peak at 3.15 Å to Cl/O ligand–ligand distances and the peak at 3.8 Å to Nb–Nb pairs in a corner-sharing geometry. Using the Cs_2_[Nb_3_O_5_Cl_7_] structure as a starting point, a cluster can be cut from the crystal structure that describes the precursor PDF. This shows that the PDF can be fitted with a polymeric Nb_*x*_Cl_*y*_O_*z*_ complex built from corner-sharing octahedra with both oxygen and chloride ligands. The structure and fit to the data are shown in Fig. 7B, with further details given in the ESI.† Some chloride atoms were substituted for oxygen atoms in order to accommodate the Nb–Cl and Nb–O peak ratio. It is here important to note that the structure models shown in Fig. 7 may not be a unique representation of the precursor clusters but contains the main structural motifs present in the precursor solutions. We expect the oxidation state of niobium to be +5 as this is oxidation state of the precursor salt (NbCl_5_) and the final product (Nb_2_O_5_). However, the oxidation state of the precursor has not been determined. It should also be noted that we cannot distinguish between O^2−^, OH^−^ or alkoxide as ligands in the complex from the X-ray PDF data. The representation given here is therefore a strong simplification of the system.

The PDFs obtained for the precursor for the 160 °C and 200 °C experiments show only very local order, and the Nb–Nb peak identified at 3.8 Å is not seen. This shows that the structures present in the precursors for these two experiments are likely isolated [NbCl_6−*x*_O_*x*_] octahedra. Note again that we cannot from the X-ray PDFs determine whether the oxygen coordinating to niobium is O^2−^, OH^−^ or an alkoxide. The precursor for the 200 °C experiment shows a higher intensity of the Nb–O peak than that for the 160 °C, suggesting a degree of Cl^−^ ligand substitution. The differences between the PDFs obtained from seemingly similar precursors are likely an effect of ageing of the precursor solution, as ligand exchange and polymerization are likely to take place already at room temperature. Substitution of chloride for oxygen ligands has previously been observed by EXAFS in a similar synthesis during the formation of tungstite in benzyl alcohol by Olliges-Stadler *et al.*^41^

The precursor structures in the 160 °C and 200 °C experiments were modelled with single octahedra models. A wavefunction was implemented to account for the exponentially damped sinusoidal oscillation caused by the interaction between niobium in the single octahedra clusters and the solvent, as previously described for nanoparticles and solvent restructuring.^62^ An illustration of the wavefunction contribution to the model is found in Fig. S14.†

### Nucleation mechanism

**Fig. 8:**
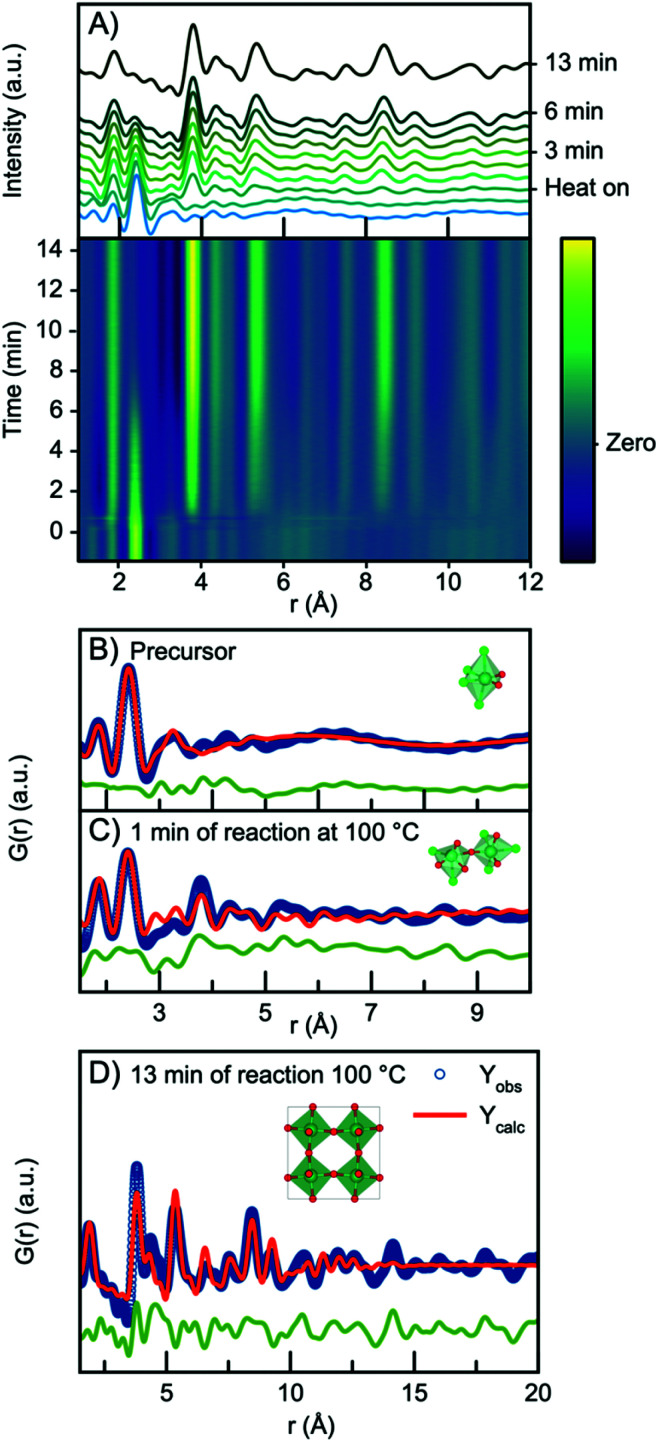
(A) Time resolved PDFs collected at ambient pressure at temperature 100 °C. (B) Refinement of the precursor PDF using a single [NbCl_4_O_2_] octahedra. (C) Refinement of the PDF collected at 1 min of reaction with a polyhedral cluster of [NbCl_6−*x*_O_*x*_] octahedra. (D) Refinement of PDF collected after 13 min of reaction using the ReO_3_ structure.

In all three experiments described above, nucleation of the crystalline phase takes place immediately after turning on the heat, and it is therefore not possible to follow the evolution from the NbCl_*x*_O_*y*_ precursor structure to the H-Nb_2_O_5_ nanoparticles within the time resolution of the experiment. We therefore conducted a similar experiment, but at 100 °C and at ambient pressure, which slowed down the reaction kinetics. The time resolved PDFs are shown in Fig. 8A.

Starting from the precursor PDF we can again identify both the Nb–O (1.8 Å) and the Nb–Cl (2.4 Å) distances as before.^60^ Compared to the precursor PDFs just investigated in Fig. 7, we observe a cluster structure similar to the two observed for the 160 °C and 200 °C experiments with no clear Nb–Nb peak. The PDF can thus be fitted with a single [NbO_2_Cl_4_] octahedra as shown in Fig. 8B. However, 1 minute into the synthesis, a clear Nb–Nb peak at 3.8 Å appears, and the PDF can be described by a polymeric cluster (Fig. 8C), similar in structure to that described above for the 300 °C precursor, where ageing and polymerization had taken place. The elevated temperature thus speeds up the polymerization we related to precursor ageing above.

As the reaction proceeds, the intensity of the first Nb–Cl peak slowly decreases, whereas the intensity of the Nb–O peak increases with time, showing that chloride is slowly substituted for oxygen. Almost simultaneously, the intensity of the first corner-sharing Nb–Nb peak increases. This is illustrated in Fig. S15,† showing the intensity of the Nb–O and Nb–Cl peak as a function of time. We can furthermore assign the next Nb–Nb peak at 5.3 Å originating from the 2^nd^ Nb–Nb distance in the ReO_3_ structure. The intensity of this peak, along with peaks at higher *r*-values, increase at almost the same rate as the first Nb–Nb peak, and the position shifts towards higher *r*-values, again illustrated in Fig. S15.† The PDFs thus show that small nanoparticles quickly form as the peaks at higher *r*-values evolve. The process illustrates that as soon as O/Cl substitution occurs, polymerization to a network of edge-sharing [NbO_6_] octahedra takes place.

The particles formed at 100 °C show almost exclusively corner-sharing octahedra as we observe no clear PDF peak at 3.3 Å, and when fitting with the simple ReO_3_ structure (Fig. 8D), the main PDF peaks can all be described, although we once again notice a clear misfit in the high *r*-region of the refinement. A similar fit (Fig. S16†) can be obtained using HNbO_3_; a hydroxide which has the same ReO_3_-type structure. The niobium hydroxide has niobium in oxidation state +5, which is chemically sensible compared to the oxidation state of +6 that the ReO_3_ structure suggests. The formation of a hydroxide structure agrees with the previously hypothesized formation mechanism for benzyl alcohol syntheses, where hydroxide species are suggested to be intermediate phases in the formation of oxide nanoparticles.^39^ The lack of a clear PDF peak from edge-sharing octahedra shows that Wadsley–Roth type structures are not suitable models for the particles formed here, and in Fig. S17,† a refinement using the H-Nb_2_O_5_ structure shows that this structure does not provide a better fit than the simple ReO_3_ structure. Implementing a second phase of R-Nb_2_O_5_ in a two-phase refinement resulted in a negative refined value of the R-Nb_2_O_5_ scale factor. The misfit of the structure above 10 Å of these small particles is thus not related to domain sizes as discussed above.

In order to investigate the nanostructure in the sample further, we conducted an additional experiment under similar conditions, which allowed *ex situ* investigations of the sample. A PDF from this sample is shown in Fig. S18,† where it agrees well with that seen from the particles formed in the *in situ* experiment, confirming reproducibility. Fig. S19† shows TEM images obtained from particles synthesized under similar conditions in a separate experiment. The images show a large degree of agglomeration of small particles on the nm scale. SAXS analysis in Fig. S20† also reflects the significant agglomeration, but a signal from small particles of *ca.* 1.6–2 nm can be identified, agreeing with the size obtained from PDF analysis. These small ReO_3_-type structures that form under ambient pressure and 100 °C are clearly related to the Wadsley–Roth particles formed at higher temperature and pressure. The ReO_3_-type network, most likely a hydroxide, can thus be seen as a nucleation cluster in the synthesis, and extended defects in the form of shear planes and Wadsley–Roth block type structures with disordered blocks are induced by temperature.

## Conclusions

Using *in situ* X-ray total scattering, we have gained atomistic insight into the formation pathway of Nb_2_O_5_ nanoparticles in the solvothermal synthesis from NbCl_5_ in benzyl alcohol. From four different *in situ* total scattering experiments conducted at four different temperatures, we show how the reaction is initialized by substitution of chloride for oxygen which leads to polymerization of [NbO_6_] octahedra. This results in the formation of small particles, highly similar in structure to ReO_3_, which can be considered nuclei for the further particle formation. These particles can be described using the HNbO_3_ niobium hydroxide structure, agreeing with previously proposed mechanisms suggested for benzyl alcohol syntheses. With growth, we observe the formation of Wadsley–Roth structured particles, which we model using the H-Nb_2_O_5_-structure. The formation of the H-Nb_2_O_5_ particles is surprising, as this phase usually forms only at elevated temperatures around 900 °C. As the particles grow further during the reaction, more edge-sharing in the structure appears, and we cannot fit the data with any of the known niobium oxide structures. However, implementing a simple R-Nb_2_O_5_ structure along with the H-Nb_2_O_5_ phase, we are able to describe the PDF peaks appearing in the higher *r*-range. We interpret this as the formation of different structure domains within the particles, and this defect chemistry appears highly dependent on particle size.

PDF analysis has thus allowed following the material all the way from NbCl_x_O_y_ precursor complexes, over HNbO_3_ hydroxide clusters, to the final, nanocrystalline niobium oxide. The type of knowledge of formation mechanisms on the atomic level gained in this study is important for the design of synthesis methods for tailor-made niobium oxide materials. While the complex chemistry of niobium oxide polymorphs means that more work is needed before the synthesis of a given Nb_2_O_5_ structure can be rationally designed, the study has provided the first insight that relates particle formation and growth to the atomic structure of Nb_2_O_5_, and opens for synthesis control of the defect chemistry in Wadsley–Roth structured phases.

## Conflicts of interest

There are no conflicts to declare.

## Supplementary Material

NR-013-D0NR08299F-s001
