# Supplementary material for: Formation and growth mechanism for niobium oxide nanoparticles: atomistic insight from in situ X-ray total scattering
Source: Nanoscale. 2021 Mar 15;13(17):8087–97. doi: 10.1039/d0nr08299f (PMC8101635; doi:10.1039/d0nr08299f)
Supplement: NR-013-D0NR08299F-s001 [file NR-013-D0NR08299F-s001.pdf]

Electronic Supplementary Material (ESI) for Nanoscale.
This journal is © The Royal Society of Chemistry 2021

SUPPORTING INFORMATION for

# Formation and growth mechanism for niobium oxide nanoparticles: Atomistic insight from *in situ* X-ray total scattering

*Olivia Aalling-Frederiksen, Mikkel Juelsholt, Andy S. Anker, and Kirsten M. Ø. Jensen**

Department of Chemistry and Nano-Science Center, University of Copenhagen

*kirsten@chem.ku.dk

## *Niobium oxide structures*

**Table S1** Overview of reported niobium oxides

| Name | Crystal system | Space group | $ReO_3$ block sizes | Formed at T (°C) from $NbO_2$[1] |
|---|---|---|---|---|
| H-$Nb_2O_5$ | Monoclinic | P 1 2/m 1 | (3x5) + (3x4) | >900 |
| M-$Nb_2O_5$ | Tetragonal | I 4/m m m | (4x4) | 850 |
| T-$Nb_2$O5 | Orthorhombic | P b a m | - | 600 - 800 |
| TT-$Nb_2O_5$ | Monoclinic/Pseudo hexagonal | - | - | 500 - 600 |
| R-$Nb_2O_5$ | Monoclinic | A 1 2/m 1 | - | |
| B-$Nb_2O_5$ | Monoclinic | B 1 1 2/b | - | |
| N-$Nb_2O_5$ | Monoclinic | C 1 2/m 1 | (4x4) | |
| P-$Nb_2O_5$ | Tetragonal | I 4 1 2 2 | - | 600 - 750 |
| $Nb_{22}O_{54}$ | Monoclinic | P 1 2/m 1 | (3x4) + (3x3) | |
| $Nb_{12}O_{29}$ | Monoclinic | A 1 m 1 | (3x4) | |

## *Background scattering signal and subtraction*

The *in situ* experiments were initiated upon applied heating. Figure S1 shows how the scattering signal from the solvent used in the experiment changes upon heating. The rapid change in the scattering pattern of the benzyl alcohol followed by a stabilization reflect that after 10 s of heating, the temperature stabilizes.

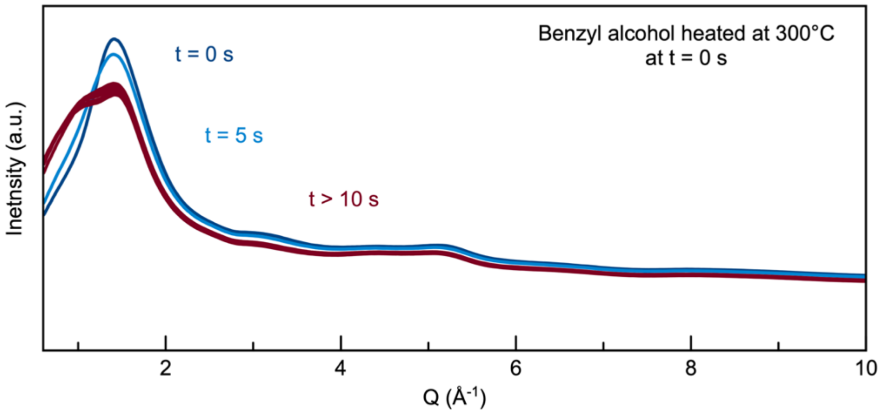


**Fig. S1** Heating of benzyl alcohol to 300 °C.

Figure S2 shows the scattering pattern obtained from the reaction solution (benzyl alcohol and $NbCl_5$) together with the background, i.e. the scattering pattern measured from the pure solvent (benzyl alcohol) in the fused silica capillary at the appropriate temperature and pressure. The scattering patterns measured from the background and precursor were first normalized to have the same intensity at the Q-max value of 14.5 $Å^{-1}$, after which a background scaling factor was determined by identifying the scaling value, where no peaks from the solvent and glass could be identified in the final PDF after background subtraction and Fourier transformation. Figure S2 shows the signal and background data obtained for frames at room temperature and after heating to 300 °C along with the final background subtracted data.

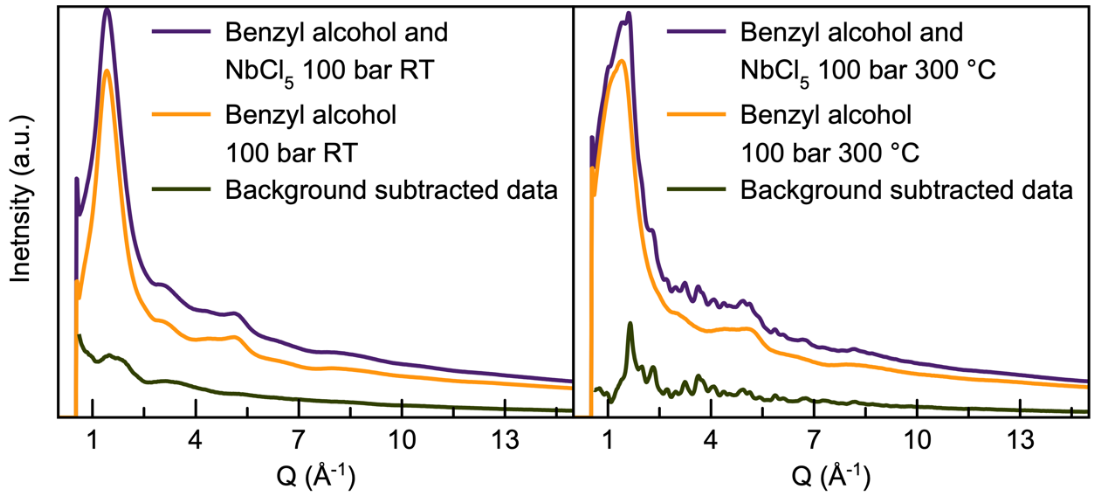


**Fig. S2** Background subtraction for the room temperature data and the data collected after 24 min of heating at 300 °C.

### ***Total scattering data and PDF analysis of $Nb_2O_5$ growth***

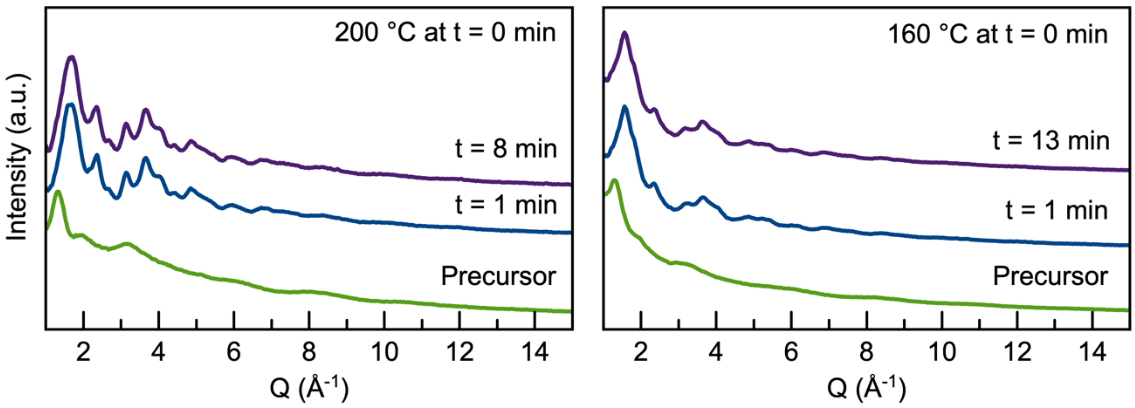


**Fig. S3** Scattering patterns collected for the experiments conducted at 160 °C and 200 °C (precursor, 1 min and 8/13 min of reaction).

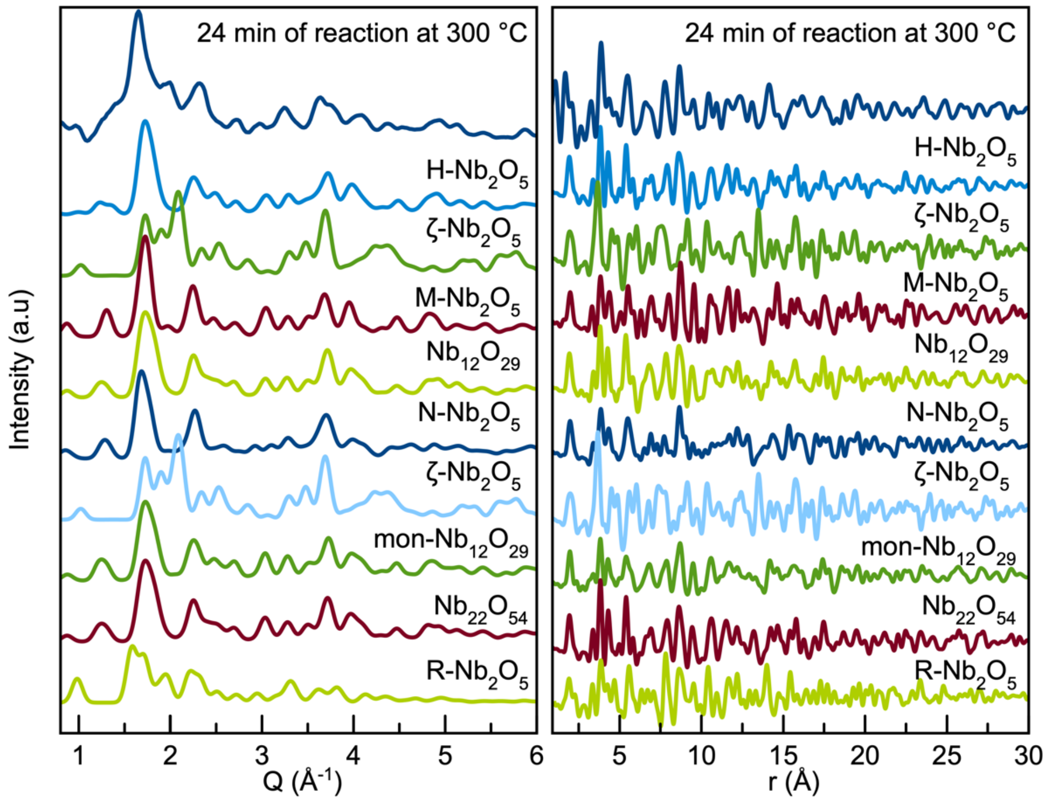


**Fig. S4** Scattering pattern and PDF collected after 24 min of reaction at 300 °C compared with calculated scattering patterns of selected niobium oxide structures. It shows that many of the niobium oxides have the same structural motif, however, none of the reported structures can fully describe the data collected.

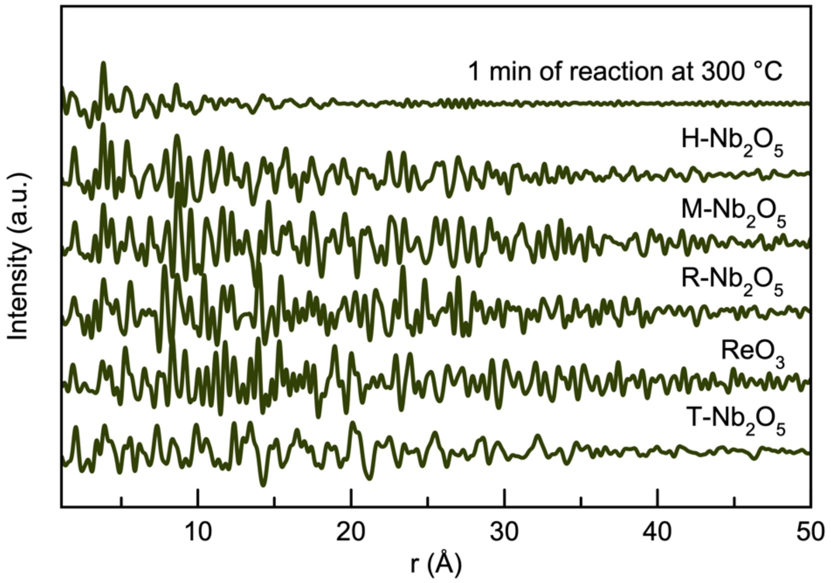


**Fig. S5** PDF collected after 1 min of reaction time at 300 °C compared with calculated PDFs of selected $Nb_2O_5$ structures and the $ReO_3$. Many of the reported structures have locally very similar PDFs.

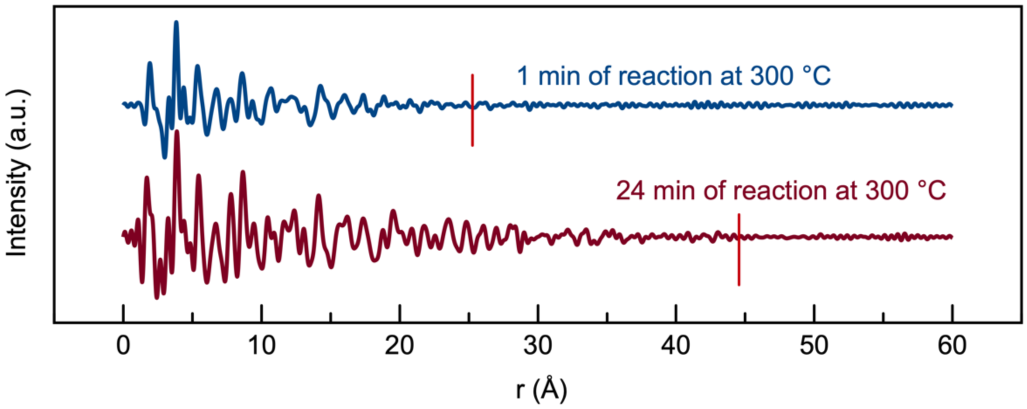


**Fig. S6** PDFs collected after 1 min and 24 min of reaction time at 300 °C showing particle growth. The PDF oscillations extend to 24 Å and 45 Å for the 1 min and 24 min data respectively.

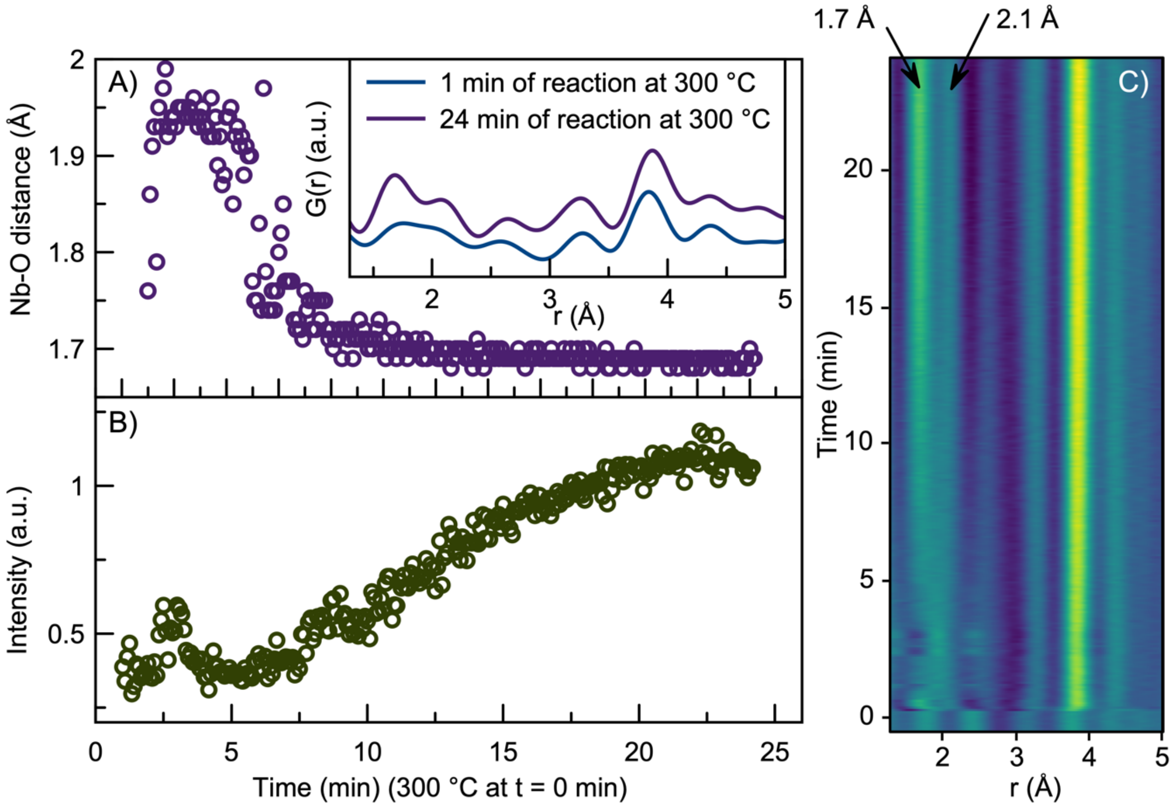


**Fig. S7** (A) First PDF peak position (Nb-O distance) plotted as a function of time. As shown from the inset there is a splitting of this peak (1.7 Å and 2.1Å). (B) The intensity of the first Nb-O distance as a function of time. The splitting is indicated by an increase in the intensity of the peak at 1.7 Å with time. (C) Contour plot showing the low *r*-range, where the peak splitting with time is clearly observed.

### *PDF refinements*

### *One phase sequential refinement, H-$Nb_2O_5$ model*

Table S2-S4 show the refinement values for datasets collected at 300 °C, 200 °C and 160 °C using the H-$Nb_2O_5$ as the structural starting model. The first frame in the sequential refinement (1 min of reaction) and the last frame were refined using all data points in the PDF. The sequential refinement of the rest of the frames was performed on the Nyquist data sampling.

The scale factor, lattice parameters a, b, c and β, $U_{iso}$ for oxygen and the sp-diameter were refined in the sequential refinement. The atomic positions of oxygen were kept fixed at the initial values obtained from the cif-file. $U_{iso}$ for niobium was furthermore fixed to a value of 0.005 Å$^2$. $\delta_2$ was kept at a value of 3 Å$^2$ which was determined from a refinement including the very low r-range. Fixed parameters were chosen in order to be able to perform a stabilized sequential refinement and keep parameters at physically reasonable values. The atomic positions of niobium were refined for the first frame in the sequential refinement (1 min of reaction) but kept fixed throughout the sequential refinement.

**Table S2** Refined values for the data collected 1 min and 24 min into the reaction at 300 °C using the H-$Nb_2O_5$ model (Fits shown in Figure 3A + B).

| | H-$Nb_2O_5$ | | |
|---|---|---|---|
| | **Initial values** | **300 °C , 1 min** | **300 °C , 24 min (last frame)** |
| **Space group** | *P* 1 2/m 1 | | |
| **Lattice par., a (Å)** | 21.153(7) | 20.84 | 20.76 |
| **Lattice par., b (Å)** | 3.8233(5) | 3.86 | 3.90 |
| **Lattice par., c (Å)** | 19.3560(50) | 19.30 | 19.29 |
| **Lattice par., β (º)** | 119.80(2) | 120.61 | 120.31 |
| **Number of refined parameters** | | 7 + 28 | 7 + 28 |
| **Data range** | | 3 Å – 30 Å | 3 Å – 30 Å |
| **$R_w$** | | 0.35 | 0.56 |
| **Scale factor** | | 0.38 | 0.52 |
| **$U_{iso}$ Nb (Å$^2$)** | | 0.005 | 0.005 |
| **$U_{iso}$ O (Å$^2$)** | | 0.044 | 0.040 |
| **$\delta_2$ (Å$^2$)** | | 3.00 | 3.00 |
| **Sp-diameter (Å)** | | 25.58 | 28.26 |

| Atomic positions for H-$Nb_2O_5$ | | | | | | |
|---|---|---|---|---|---|---|
| | **Initial values** | | | **Refined values** | | |
| **Label** | x (Å) | y (Å) | z (Å) | x (Å) | y (Å) | z (Å) |
| **Nb1** | 0 | 0.2285(3) | 0 | 0.000000 | 0.190820 | 0.000000 |
| **Nb2** | 0.5 | 0 | 0.5 | 0.500000 | 0.000000 | 0.500000 |
| **Nb3** | 0.16430(3) | 0 | 0.00353(3) | 0.156992 | 0.000000 | 0.000249 |
| **Nb4** | 0.23623(3) | 0 | 0.23134(3) | 0.241400 | 0.000000 | 0.231220 |
| **Nb5** | 0.30284(3) | 0 | 0.45456(3) | 0.299751 | 0.000000 | 0.457878 |
| **Nb6** | 0.36087(3) | 0 | 0.04553(3) | 0.360465 | 0.000000 | 0.056640 |
| **Nb7** | 0.43325(3) | 0 | 0.27741(4) | 0.445501 | 0.000000 | 0.280925 |
| **Nb8** | 0.56270(3) | 0 | 0.09346(3) | 0.555657 | 0.000000 | 0.086263 |
| **Nb9** | 0.62943(3) | 0 | 0.32219(3) | 0.640662 | 0.000000 | 0.313702 |
| **Nb10** | 0.09353(3) | 0.5 | 0.20187(3) | 0.101029 | 0.500000 | 0.205564 |
| **Nb11** | 0.15921(3) | 0.5 | 0.42410(3) | 0.152594 | 0.500000 | 0.420687 |
| **Nb12** | 0.70330(3) | 0.5 | 0.12414(3) | 0.698531 | 0.500000 | 0.129695 |
| **Nb13** | 0.77056(3) | 0.5 | 0.35151(3) | 0.778609 | 0.500000 | 0.350835 |
| **Nb14** | 0.89895(3) | 0.5 | 0.16353(3) | 0.901519 | 0.500000 | 0.151909 |
| **Nb15** | 0.96461(3) | 0.5 | 0.38928(4) | 0.982518 | 0.500000 | 0.405926 |

**Table S3** Refined values for the data collected 1 min and 5 min into the reaction at 200 °C using the H-$Nb_2O_5$ model (Fit shown in Figure 6D).

| | H-$Nb_2O_5$ | | |
|---|---|---|---|
| | **Initial values** | **200 °C, 1 min** | **200 °C, 5 min (last frame)** |
| **Space group** | *P* 1 2/m 1 | | |
| **Lattice par., a (Å)** | 21.153(7) | 20.83 | 20.89 |
| **Lattice par., b (Å)** | 3.8233(5) | 3.87 | 3.87 |
| **Lattice par., c (Å)** | 19.3560(50) | 19.77 | 19.67 |
| **Lattice par., β (°)** | 119.80(2) | 118.75 | 118.67 |
| **Number of refined parameters** | | 7 + 28 | 7 + 28 |
| **Data range** | | 3 Å – 30 Å | 3 Å – 30 Å |
| **$R_w$** | | 0.35 | 0.40 |
| **Scale factor** | | 0.42 | 0.47 |
| **$U_{iso}$ Nb (Å$^2$)** | | 0.005 | 0.005 |
| **$U_{iso}$ O (Å$^2$)** | | 0.070 | 0.077 |
| **$\delta_2$ (Å$^2$)** | | 3.00 | 3.00 |
| **Sp-diameter (Å)** | | 20.67 | 20.28 |

| Atomic positions for H-$Nb_2O_5$ | | | | | | |
|---|---|---|---|---|---|---|
| | **Initial values** | | | **Refined values** | | |
| **Label** | x (Å) | y (Å) | z (Å) | x (Å) | y (Å) | z (Å) |
| **Nb1** | 0 | 0.2285(3) | 0 | 0.000000 | 0.012848 | 0.000000 |
| **Nb2** | 0.5 | 0 | 0.5 | 0.500000 | 0.000000 | 0.500000 |
| **Nb3** | 0.16430(3) | 0 | 0.00353(3) | 0.164827 | 0.000000 | 0.011097 |
| **Nb4** | 0.23623(3) | 0 | 0.23134(3) | 0.212939 | 0.000000 | 0.197181 |
| **Nb5** | 0.30284(3) | 0 | 0.45456(3) | 0.304924 | 0.000000 | 0.450204 |
| **Nb6** | 0.36087(3) | 0 | 0.04553(3) | 0.365570 | 0.000000 | 0.049181 |
| **Nb7** | 0.43325(3) | 0 | 0.27741(4) | 0.440342 | 0.000000 | 0.279791 |
| **Nb8** | 0.56270(3) | 0 | 0.09346(3) | 0.556894 | 0.000000 | 0.092212 |
| **Nb9** | 0.62943(3) | 0 | 0.32219(3) | 0.642206 | 0.000000 | 0.338850 |
| **Nb10** | 0.09353(3) | 0.5 | 0.20187(3) | 0.085348 | 0.500000 | 0.192639 |
| **Nb11** | 0.15921(3) | 0.5 | 0.42410(3) | 0.145172 | 0.500000 | 0.414201 |
| **Nb12** | 0.70330(3) | 0.5 | 0.12414(3) | 0.693303 | 0.500000 | 0.132323 |
| **Nb13** | 0.77056(3) | 0.5 | 0.35151(3) | 0.757023 | 0.500000 | 0.321090 |
| **Nb14** | 0.89895(3) | 0.5 | 0.16353(3) | 0.890187 | 0.500000 | 0.137560 |
| **Nb15** | 0.96461(3) | 0.5 | 0.38928(4) | 0.963236 | 0.500000 | 0.403199 |

**Table S4** Refined values for the data collected 1 min and 13 min into the reaction at 1600 °C using the H-$Nb_2O_5$ model (Fit shown in Figure 6C).

| | **H-$Nb_2O_5$** | | |
|---|---|---|---|
| | **Initial values** | **160 °C, 1 min** | **160 °C, 13 min (last frame)** |
| **Space group** | *P* 1 2/m 1 | | |
| **Lattice par., a (Å)** | 21.153(7) | 20.77 | 20.97 |
| **Lattice par., b (Å)** | 3.8233(5) | 3.77 | 3.78 |
| **Lattice par., c (Å)** | 19.3560(50) | 19.76 | 19.86 |
| **Lattice par., β (°)** | 119.80(2) | 121.85 | 121.89 |
| **Number of refined parameters** | | 7 + 28 | 7 + 28 |
| **Data range** | | 3 Å – 20 Å | 3 Å – 20 Å |
| **$R_w$** | | 0.42 | 0.40 |
| **Scale factor** | | 0.21 | 0.17 |
| **$U_{iso}$ Nb (Å$^2$)** | | 0.005 | 0.005 |
| **$U_{iso}$ O (Å$^2$)** | | 0.0029 | 0.0022 |
| **$\delta_2$ (Å$^2$)** | | 3.00 | 3.00 |
| **Sp-diameter (Å)** | | 12.86 | 14.63 |

| **Atomic positions for H-$Nb_2O_5$** | | | | | | |
|---|---|---|---|---|---|---|
| | **Initial values** | | | **Refined values** | | |
| **Label** | x (Å) | y (Å) | z (Å) | x (Å) | y (Å) | z (Å) |
| **Nb1** | 0 | 0.2285(3) | 0 | 0.000000 | 0.117279 | 0.000000 |
| **Nb2** | 0.5 | 0 | 0.5 | 0.500000 | 0.000000 | 0.500000 |
| **Nb3** | 0.16430(3) | 0 | 0.00353(3) | 0.162612 | 0.000000 | 0.017641 |
| **Nb4** | 0.23623(3) | 0 | 0.23134(3) | 0.239352 | 0.000000 | 0.235610 |
| **Nb5** | 0.30284(3) | 0 | 0.45456(3) | 0.310369 | 0.000000 | 0.460093 |
| **Nb6** | 0.36087(3) | 0 | 0.04553(3) | 0.353042 | 0.000000 | 0.051398 |
| **Nb7** | 0.43325(3) | 0 | 0.27741(4) | 0.439332 | 0.000000 | 0.279767 |
| **Nb8** | 0.56270(3) | 0 | 0.09346(3) | 0.550582 | 0.000000 | 0.102557 |
| **Nb9** | 0.62943(3) | 0 | 0.32219(3) | 0.646236 | 0.000000 | 0.327372 |
| **Nb10** | 0.09353(3) | 0.5 | 0.20187(3) | 0.095710 | 0.500000 | 0.198541 |
| **Nb11** | 0.15921(3) | 0.5 | 0.42410(3) | 0.143072 | 0.500000 | 0.413255 |
| **Nb12** | 0.70330(3) | 0.5 | 0.12414(3) | 0.689860 | 0.500000 | 0.135442 |
| **Nb13** | 0.77056(3) | 0.5 | 0.35151(3) | 0.763397 | 0.500000 | 0.365031 |
| **Nb14** | 0.89895(3) | 0.5 | 0.16353(3) | 0.901001 | 0.500000 | 0.176405 |
| **Nb15** | 0.96461(3) | 0.5 | 0.38928(4) | 0.955898 | 0.500000 | 0.387857 |

### *One-phase sequential refinement, $Nb_{12}O_{29}$ and $Nb_{22}O_{54}$ model*

Sequential refinements using other niobium oxides show how other structures with the same local motifs give highly similar results compared to the refinements using the H-$Nb_2O_5$ model.
In the refinement using $Nb_{12}O_{29}$ as the starting model a scale factor, lattice parameters a, b, c and β, $U_{iso}$ for oxygen and niobium and the sp-diameter were refined in the sequential refinement. The niobium positions were refined for the first frame (1 min of reaction) and kept fixed in the sequential refinement.
In the refinement using $Nb_{22}O_{29}$ as the structural starting model a scale factor, lattice parameters a, b, c and β, $U_{iso}$ for oxygen and niobium and the sp-diameter were refined in the sequential refinement.

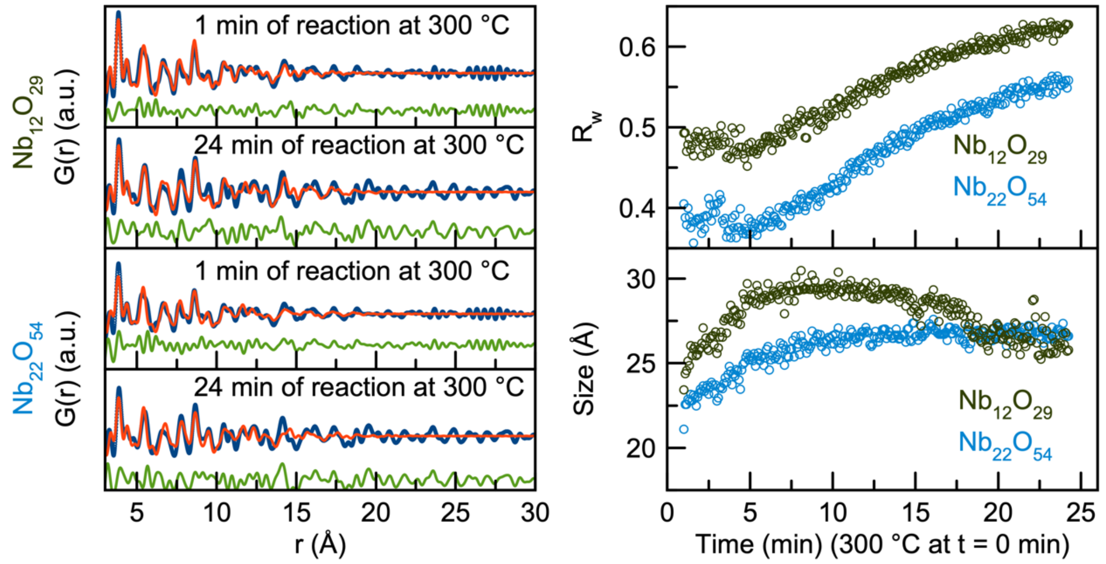


**Fig. S8** Sequential refinement of data collected at 300 °C using $Nb_{12}O_{29}$ and $Nb_{22}O_{54}$ respectively as the structure model. The changes in both $R_w$ and sp-diameter with time look highly similar to those observed in the refinement using the H-$Nb_2O_5$ model

**Table S5** Refined values for data collected 1 min and 24 min of reaction at 300 °C using $Nb_{12}O_{29}$ as the structural starting model (Figure S8).

| | $Nb_{12}O_{29}$ | | |
|---|---|---|---|
| | **Initial values** | **300 °C , 1min** | **300 °C , 24 min (last frame)** |
| **Space group** | *P* 1 2/m 1 | | |
| **Lattice par., a (Å)** | 15.6856 | 15.56 | 15.50 |
| **Lattice par., b (Å)** | 3.8307 | 3.85 | 3.87 |
| **Lattice par., c (Å)** | 20.71 | 20.52 | 20.59 |
| **Lattice par., β (°)** | 113.056 | 113.49 | 113.78 |
| **Cell vol., V (Å$^3$)** | | | |
| **Number of refined parameters** | | 20 | 8 + 12 |
| **Data range** | | 3 Å – 30 Å | 3 Å – 30 Å |
| **$R_w$** | | 0.41 | 0.56 |
| **Scale factor** | | 0.39 | 0.51 |
| **$U_{iso}$ Nb (Å$^{-2}$)** | | 0.0032 | 0.0042 |
| **$U_{iso}$ O (Å$^{-2}$)** | | 0.015 | 0.016 |
| **Sp-diameter (Å)** | | 21.10 | 25.58 |

| Atomic positions for $Nb_{12}O_{29}$ | | | | | | |
|---|---|---|---|---|---|---|
| | Initial values | | | Refined values | | |
| **Label** | x (Å) | y (Å) | z (Å) | x (Å) | y (Å) | z (Å) |
| **Nb1** | 0.1004(9) | 0 | 0.0689(7) | 0.092171 | 0.000000 | 0.071936 |
| **Nb2** | 0.0997(9) | 0 | 0.6996(7) | 0.097270 | 0.000000 | 0.699624 |
| **Nb3** | 0.0985(9) | 0 | 0.8822(6) | 0.103033 | 0.000000 | 0.887738 |
| **Nb4** | 0.373(1) | 0 | 0.1455(7) | 0.366071 | 0.000000 | 0.141586 |
| **Nb5** | 0.367(1) | 0 | 0.7746(7) | 0.375591 | 0.000000 | 0.775090 |
| **Nb6** | 0.365(1) | 0 | 0.958(1) | 0.369910 | 0.000000 | 0.958615 |

**Table S6** Refined values for data collected 1 min and 24 min of reaction at 300 °C using $Nb_{22}O_{54}$ as the structural starting model (Figure S8).

| | $Nb_{22}O_{54}$ | | |
|---|---|---|---|
| | **Initial values** | **300 °C, 1 min** | **300 °C, 24 min (last frame)** |
| **Space group** | *P* 1 2/m 1 | | |
| **Lattice par., a (Å)** | 15.7491 | 15.45 | 15.81 |
| **Lattice par., b (Å)** | 3.8236 | 3.85 | 3.88 |
| **Lattice par., c (Å)** | 17.8521 | 17.95 | 17.69 |
| **Lattice par., β (°)** | 102.029 | 103.62 | 102.75 |
| **Cell vol., V (Å$^3$)** | | | |
| **Number of refined parameters** | | 8 | 8 |
| **Data range** | | 3 Å – 30 Å | 3 Å – 30 Å |
| **$R_w$** | | 0.49 | 0.63 |
| **Scale factor** | | 0.35 | 0.46 |
| **$U_{iso}$ Nb (Å$^{-2}$)** | | 0.0082 | 0.0075 |
| **$U_{iso}$ O (Å$^{-2}$)** | | 0.033 | 0.034 |
| **Sp-diameter (Å)** | | 23.38 | 25.54 |

## *Refinement of the data collected 24 min of reaction at 300 °C, $ReO_3$ and R-$Nb_2O_5$ model*

**Table S7** Refined values for data collected 24 min into the reaction at 300 °C using $ReO_3$ as the structural model (Figure 5A).

| | Initial values | 300 °C, 24 min (last frame) |
|---|---|---|
| | $ReO_3$ | |
| **Space group** | *I* m -3 | |
| **Lattice par., a = b = c (Å)** | 7.4456(2) | 7.74 |
| **Number of refined parameters** | | 5 |
| **Data range** | | 3 Å – 30 Å |
| **$R_w$** | | 0.68 |
| **Scale factor** | | 0.25 |
| **$U_{iso}$ Nb ($Å^{-2}$)** | | 0.012 |
| **$U_{iso}$ O ($Å^{-2}$)** | | 0.11 |
| **$\delta_2$ ($Å^2$)** | | 3.00 |
| **Sp-diameter (Å)** | | 23.64 |

**Table S8** Refined values with data collected 24 min into the reaction at 300 °C using $ReO_3$ and R-$Nb_2O_5$ as the structural models (Figure 5B).

| | Initial values | 300 °C, 24 min (last frame) |
|---|---|---|
| | $ReO_3$ | |
| **Space group** | *I* m -3 | |
| **Lattice par., a = b = c (Å)** | 7.4456(2) | 7.68 |
| **Number of refined parameters** | | 10 |
| **Data range** | | 3 Å – 30 Å |
| **$R_w$** | | 0.47 |
| **Scale factor** | | 0.069 |
| **$U_{iso}$ Nb ($Å^{-2}$)** | | 0.010 |
| **$U_{iso}$ O ($Å^{-2}$)** | | 0.12 |
| **$\delta_2$ ($Å^2$)** | | 3.00 |
| **Sp-diameter (Å)** | | 42.01 |
| | R-$Nb_2O_5$ | |
| **Space group** | | A 1 2/m 1 |
| **Lattice par., a (Å)** | 3.983(4) | 3.89 |
| **Lattice par., b (Å)** | 3.826(4) | 3.89 |
| **Lattice par., c (Å)** | 12.79(1) | 12.90 |
| **Lattice par., β (°)** | 90.75(5) | 92.04 |
| **Scale** | | 0.28 |

### *Two-phase sequential refinement with R-$Nb_2O_5$ and H-$Nb_2O_5$*

The data collected at 300 °C were refined using a two-phase refinement with H-$Nb_2O_5$ and R-$Nb_2O_5$. Both $U_{iso}$ for niobium and oxygen along with the sp-diameter were constrained to the same values for both phases throughout the sequential refinement.

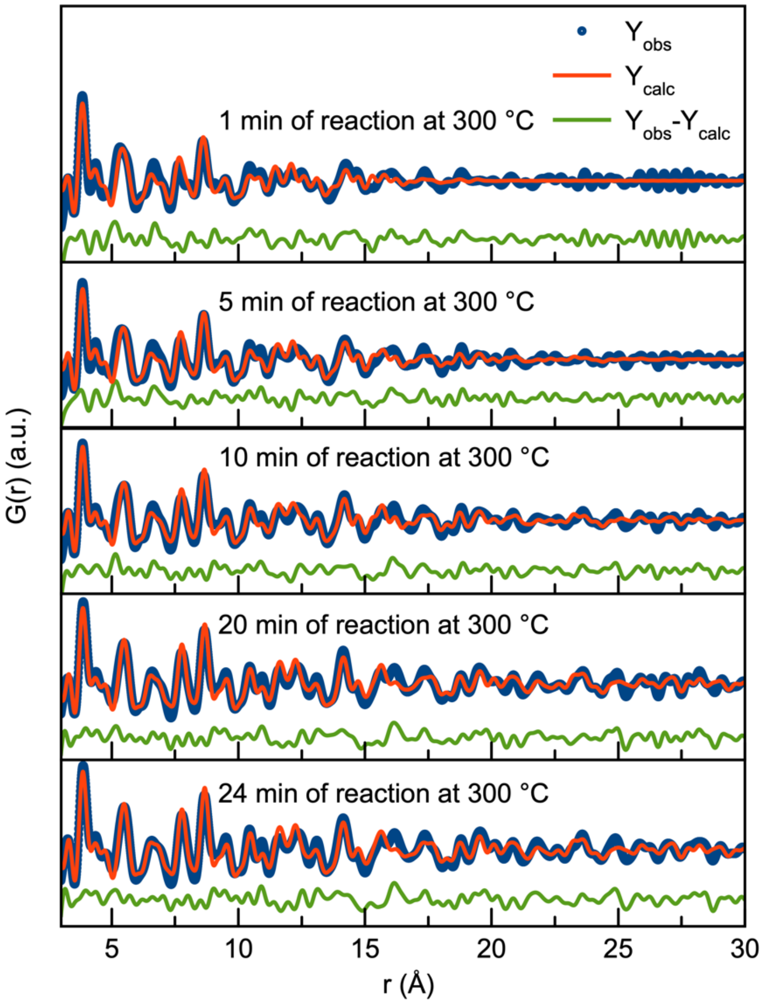


**Fig. S9** Selected fits from the sequential refinement of the data collected at 300 °C using H- and R-$Nb_2O_5$ as the structural starting models.

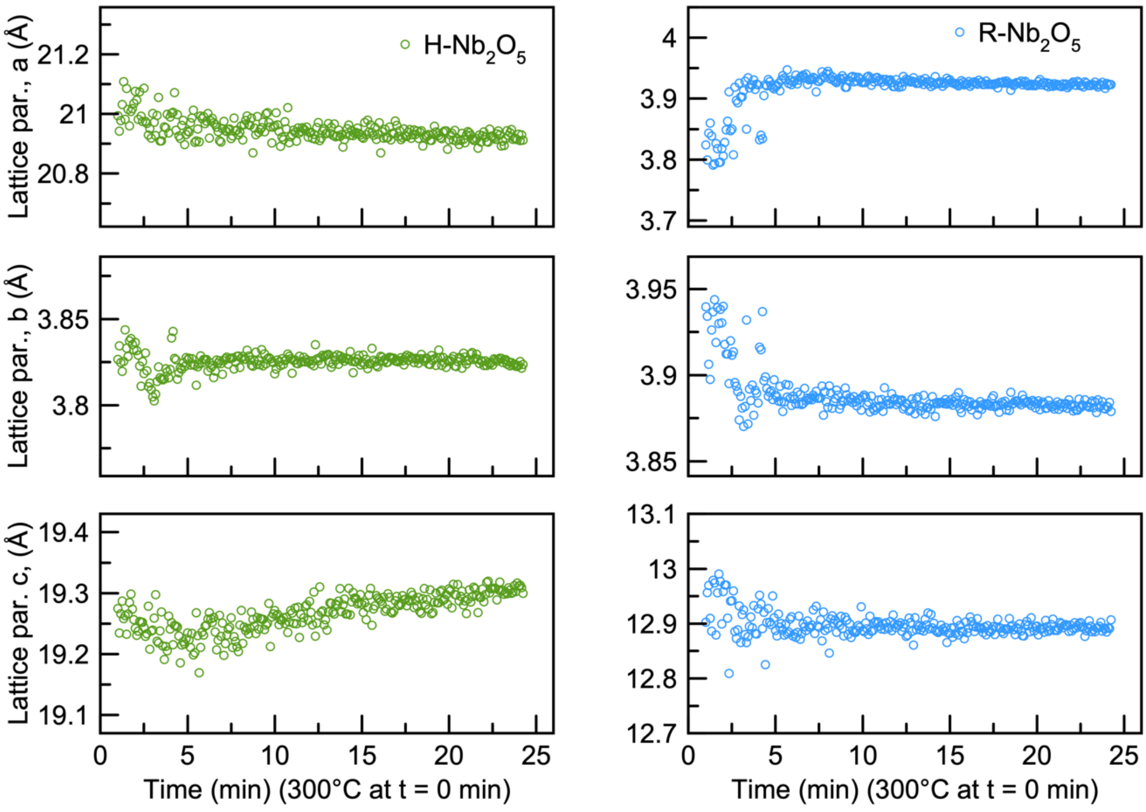


**Fig. S10** Lattice parameters as a function of time from the two-phase refinement of the data collected at 300 °C using H- and R-$Nb_2O_5$ as models.

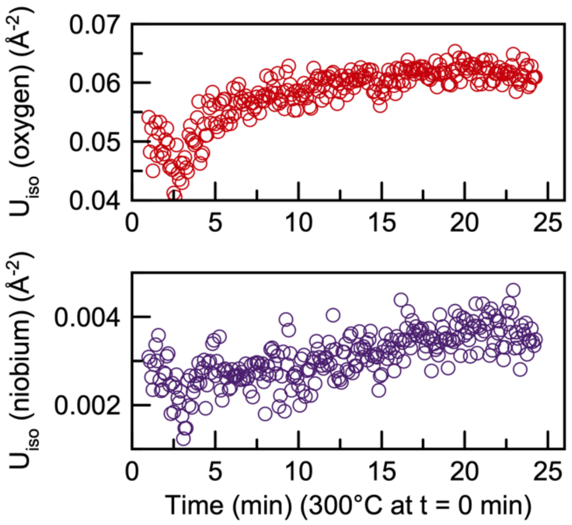


**Fig. S11** $U_{iso}$ for niobium and oxygen as a function of time from the two-phase refinement of the data collected at 300 °C using H- and R-$Nb_2O_5$ as models.

**Table S9** Refined values with data collected at different time-points in the synthesis 300 °C using H-$Nb_2O_5$ and R-$Nb_2O_5$ as the structural starting models (Figure 5C + S9).

| | Initial values | 1 min | 5 min | 10 min | 20 min | 24 min |
|---|---|---|---|---|---|---|
| | **H-$Nb_2O_5$** | | | | | |
| **Space group** | *P* 1 2/m 1 | | | | | |
| **Lattice par., a (Å)** | 21.153(7) | 20.99 | 20.94 | 20.96 | 20.91 | 20.91 |
| **Lattice par., b (Å)** | 3.8233(5) | 3.83 | 3.83 | 3.82 | 3.83 | 3.82 |
| **Lattice par., c (Å)** | 19.3560(50) | 19.27 | 19.23 | 19.27 | 19.31 | 19.30 |
| **Lattice par., β (°)** | 119.80(2) | 120.44 | 120.22 | 120.33 | 120.33 | 120.29 |
| | **R-$Nb_2O_5$** | | | | | |
| **Space group** | A 1 2/m 1 | | | | | |
| **Lattice par., a (Å)** | 3.983(4) | 3.82 | 3.92 | 3.93 | 3.92 | 3.92 |
| **Lattice par., b (Å)** | 3.826(4) | 3.94 | 3.89 | 3.89 | 3.88 | 3.88 |
| **Lattice par., c (Å)** | 12.79(1) | 12.90 | 12.89 | 12.89 | 12.90 | 12.91 |
| **Lattice par., β (°)** | 90.75(5) | 92.89 | 91.52 | 91.66 | 91.53 | 91.59 |
| **Number of refined parameters** | | 13 + 29 | 13 + 29 | 13 + 29 | 13 + 29 | 13 + 29 |
| **Data range** | | 3 Å – 30 Å | 3 Å – 30 Å | 3 Å – 30 Å | 3 Å – 30 Å | 3 Å – 30 Å |
| **$R_w$** | | 0.45 | 0.40 | 0.36 | 0.35 | 0.35 |
| **Scale factor (H)** | | 0.42 | 0.35 | 0.31 | 0.29 | 0.29 |
| **Scale factor (R)** | | 0.062 | 0.091 | 0.13 | 0.17 | 0.18 |
| **$U_{iso}$ Nb ($Å^{-2}$)** | | 0.0031 | 0.0027 | 0.0024 | 0.0036 | 0.0035 |
| **$U_{iso}$ O ($Å^{-2}$)** | | 0.062 | 0.053 | 0.059 | 0.064 | 0.061 |
| **Sp-diameter (Å)** | | 22.70 | 31.74 | 40.28 | 53.66 | 53.50 |

| **Atomic positions for H-$Nb_2O_5$** | | | | | | |
|---|---|---|---|---|---|---|
| | **Initial values** | | | **Refined values** | | |
| | x (Å) | y (Å) | z (Å) | x (Å) | y (Å) | z (Å) |
| **Nb1** | 0 | 0.2285(3) | 0 | 0.000000 | 0.181599 | 0.000000 |
| **Nb2** | 0.5 | 0 | 0.5 | 0.500000 | 0.000000 | 0.500000 |
| **Nb3** | 0.16430(3) | 0 | 0.00353(3) | 0.155071 | 0.000000 | -0.011129 |
| **Nb4** | 0.23623(3) | 0 | 0.23134(3) | 0.241870 | 0.000000 | 0.236585 |
| **Nb5** | 0.30284(3) | 0 | 0.45456(3) | 0.293222 | 0.000000 | 0.463501 |
| **Nb6** | 0.36087(3) | 0 | 0.04553(3) | 0.379844 | 0.000000 | 0.047979 |
| **Nb7** | 0.43325(3) | 0 | 0.27741(4) | 0.438832 | 0.000000 | 0.283428 |
| **Nb8** | 0.56270(3) | 0 | 0.09346(3) | 0.567540 | 0.000000 | 0.083822 |
| **Nb9** | 0.62943(3) | 0 | 0.32219(3) | 0.637105 | 0.000000 | 0.307446 |
| **Nb10** | 0.09353(3) | 0.5 | 0.20187(3) | 0.109324 | 0.500000 | 0.189915 |
| **Nb11** | 0.15921(3) | 0.5 | 0.42410(3) | 0.158402 | 0.500000 | 0.421711 |
| **Nb12** | 0.70330(3) | 0.5 | 0.12414(3) | 0.694720 | 0.500000 | 0.119776 |
| **Nb13** | 0.77056(3) | 0.5 | 0.35151(3) | 0.771249 | 0.500000 | 0.351663 |
| **Nb14** | 0.89895(3) | 0.5 | 0.16353(3) | 0.880633 | 0.500000 | 0.164061 |
| **Nb15** | 0.96461(3) | 0.5 | 0.38928(4) | 0.968163 | 0.500000 | 0.387467 |
| **Atomic positions for R-$Nb_2O_5$** | | | | | | |
| **Nb1** | 0.07(1) | 0 | 0.146(2) | 0.051956 | 0.00000 | 0.146677 |

## *One-phase sequential refinement of 160 °C and 200 °C with H-$Nb_2O_5$*

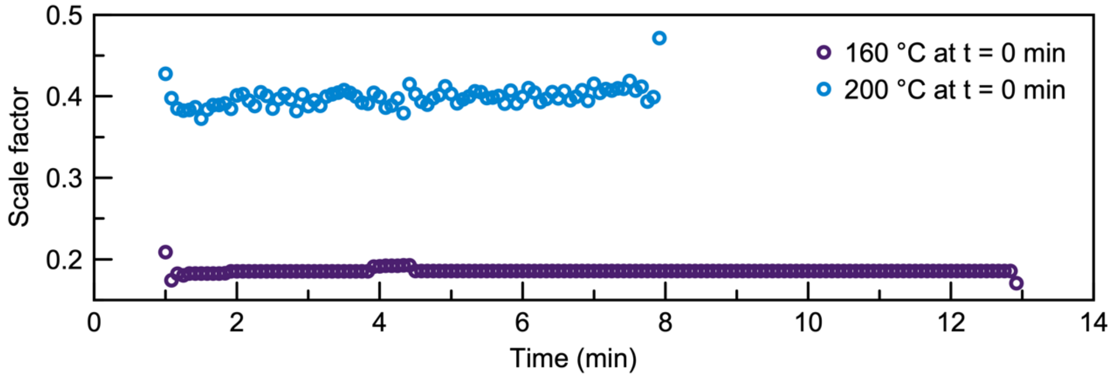


**Fig. S12** From sequential refinement of the data collected at 160 °C and 200 °C with H-$Nb_2O_5$ we observe no significant changes in the scale factors.

## *Cluster refinement of precursor PDFs*

For the refinement of the precursor PDFs we apply *Diffpy-CMI*[2] and perform a least-square optimization between a theoretical calculated PDF (calculated with the Debye equation) and the experimental PDF. The cluster models used for the calculated PDF were all obtained as cutouts from the $Cs_2[Nb_3O_5Cl_7]$ crystal structure shown in Figure S12. Clusters with different sizes and different Cl/O ratios were extracted in order to get the best structural starting models for the refinements. Figures S12 also shows such a cut-out.
In all refinements, the scale factor and Nb atomic positions were refined. The isotropic ADP values of Cl, Nb and O were all fixed to 0.03 Å$^2$. For the refinement with single octahedra, a wavefunction was implemented in order to describe the solvent-cluster interaction.[3]

$$w(r) = A \cdot sin\left(2\pi\left(\frac{r}{\lambda} - \varphi\right)\right) e^{-\left(\frac{(r-r_0)}{2\sigma_{eff}}\right)^2}$$

Figure S13 shows the refinement of the precursor data for the 200°C experiment both with a without implementing the wave function.

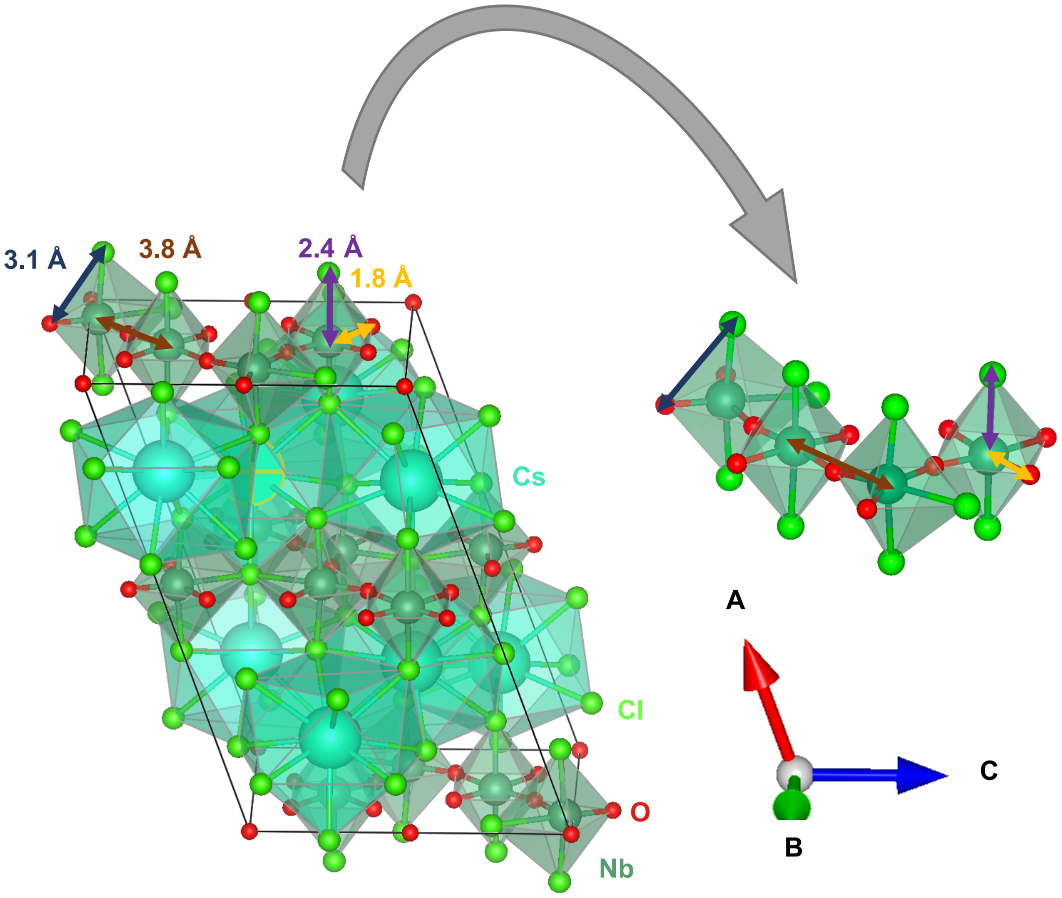


**Fig. S13** Crystal structure of $Cs_2[Nb_3O_5Cl_7]$ to the left. Bright green: Cl, dark green: Nb, turquoise: Cs and red: O. A cut-out illustrating how the models for the refinement were extracted.

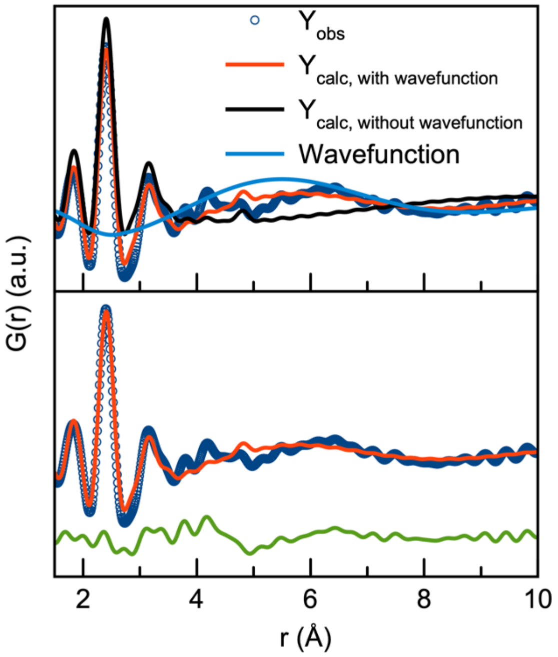


**Fig. S14** Refinement of the precursor PDF collected for the 160 °C experiment using a single $[NbCl_{6-x}O_x]$ octahedra showing the contribution from the wavefunction.

**Table S10** Refined values of the precursor collected for the experiment conducted at 300 °C (Figure 7B). Here, a chain of four [$NbCl_{6-x}O_x$] was used in the structural refinement.

| | **Initial values** | **Precursor for the 300 °C experiment** |
|---|---|---|
| **Nb0 position (x,y,z)** | (-7.22, 7.05, 2.07) | (-7.10, 7.00, 2.06) |
| **Nb1 position (x,y,z)** | (-5.38, 1.88, 1.54) | (-5.43, 1.89, 1.54) |
| **Nb2 position (x,y,z)** | (-6.43, 4.18, 1.83) | (-6.33, 4.28, 1.83) |
| **Nb3 position (x,y,z)** | (-4.74, 4.74, 1.31) | (-4.70, 4.60, 1.30) |
| **Data range** | | 1.5 Å – 10 Å |
| **Data summation number** | | 1 frame (5 s) |
| **Number of refined parameters** | | 13 |
| **Scale** | | 0.25 |
| **$R_w$** | | 0.55 |

**Table S11** Refined values of the precursor collected for the experiment conducted at 200 °C and 160 °C. Here single [$NbCl_{6-x}O_x$] octahedra were used in the structural refinements (Figure 7C + D)

| | Initial values | Precursor for the 200 °C experiment | Precursor for the 160 °C experiment |
|---|---|---|---|
| **Nb0 position (x,y,z)** | (1.45, 4.22, 7.83) | (1.45, 4.28, 7.89) | (1.46, 4.20, 7.87) |
| **Data range** | | 1.5 Å – 10 Å | 1.5 Å – 10 Å |
| **Data summation number** | | 6 frames (30 s) | 58 frames (290 s) |
| **Number of refined parameters** | | 10 | 10 |
| **Scale** | | 0.346 | 0.207 |
| **$R_w$** | | 0.349 | 0.324 |
| **wA** | | -0.28 | 110 |
| **wasym** | | 3.73 | 2.50 |
| **wlam** | | 7.03 | 5.92 |
| **wphi** | | 1.03 | 7.15 |
| **wr0** | | -4.08 | -8.94 |
| **wsig** | | -1.02 | 7.10 |

## ***Data analysis of experiment conducted at 100 °C with ambient pressure***

In order to slow down the reaction an experiment at 100 °C and ambient pressure was conducted.

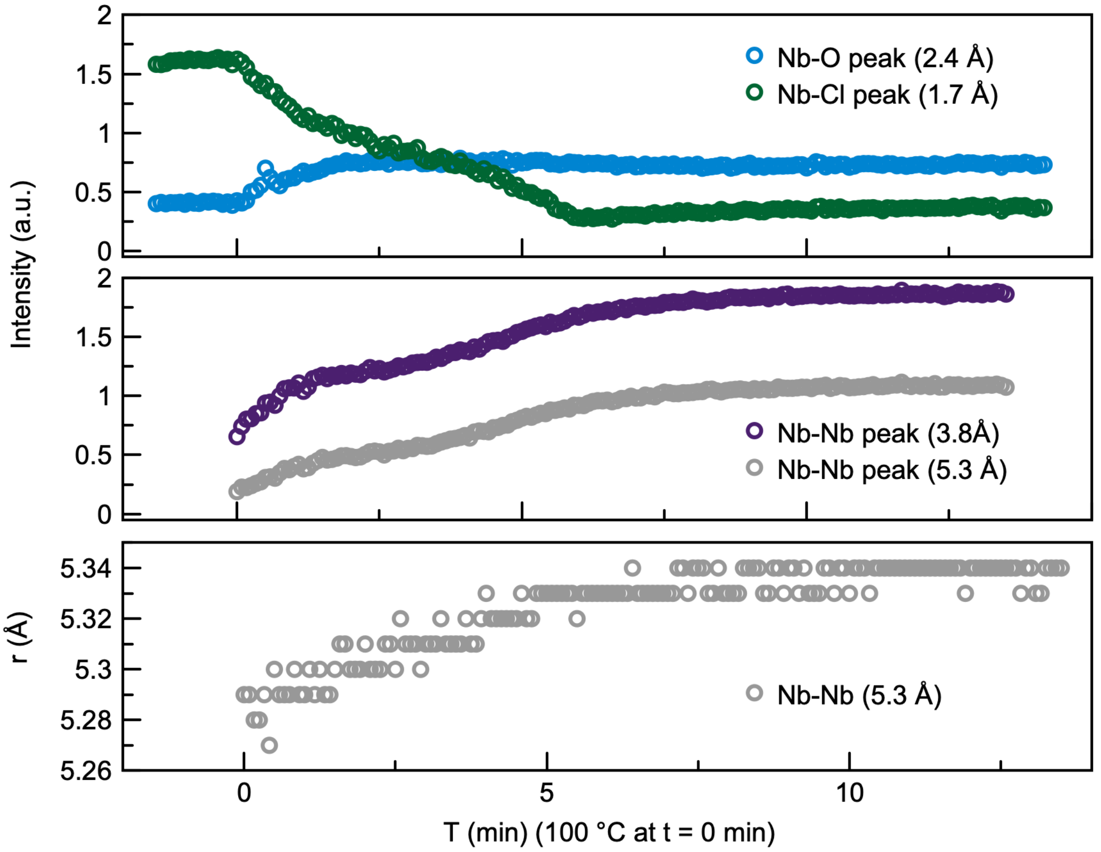


**Fig. S15** Changes in intensity and peak positions of selected structural peaks plotted as a function of time of the reaction in the experiment conducted at 100 °C.

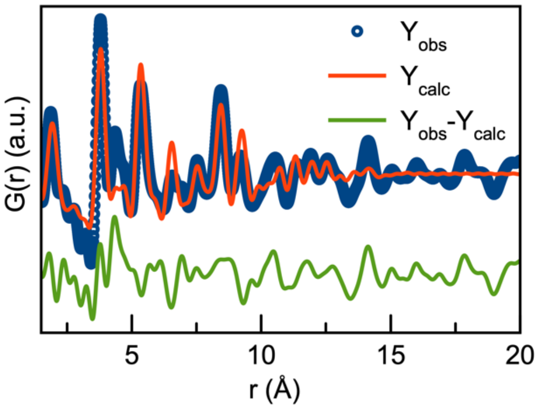


**Fig. S16** Structural refinement of data collected after 13 min of reaction at 100 °C using the structural model of $HNbO_3$.

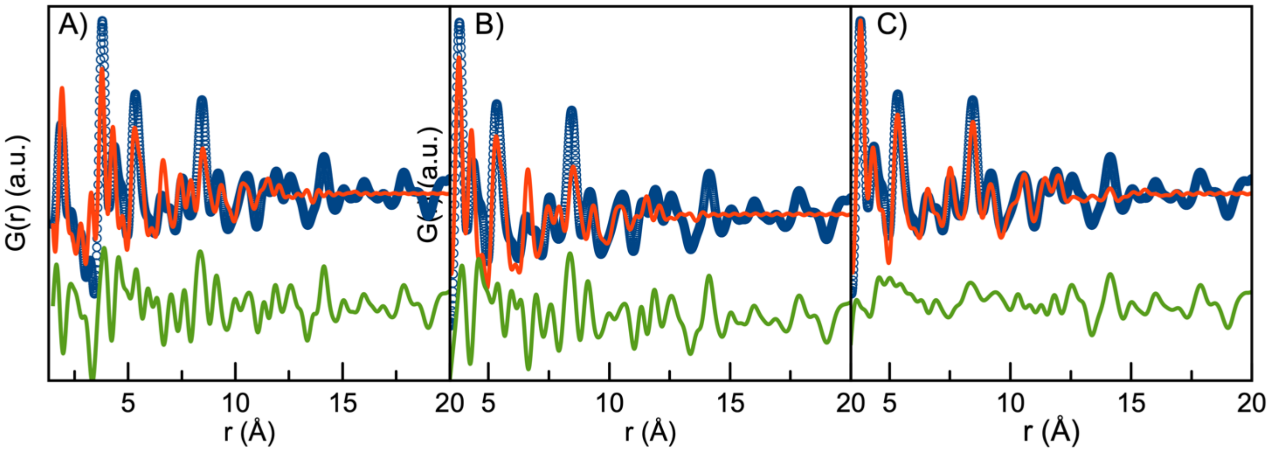


**Fig. S17** PDF refinement of data collected at 13 min of reaction 100 °C and ambient pressure using H-$Nb_2O_5$ as the structural starting model. (A) Without refining niobium atomic positions including the first Nb-O peak. (B) Without the refinement of niobium atomic positions. (C) With the refinement of the niobium atomic positions.

**Table S12** Refined values for data collected at 100 °C using a single $[NbCl_{6-x}O_x]$ octahedra for the precursor data and a chain of two $[NbCl_{6-x}O_x]$ octahedra for the data collected after 1 min of reaction (Figure 8B + C).

| | **Initial values** | **Precursor** | **Initial values** | **100 °C , 1 min** |
|---|---|---|---|---|
| **Nb0 position (x,y,z)** | (1.45, 4.18, 7.85) | (1.44, 4.19, 7.92) | (15.3, 7.08, 5.47) | (15.3, 6.99, 5.45) |
| **Nb1 position (x,y,z)** | | | (14.5, 4.20, 7.79) | (14.5, 4.20, 7.83) |
| **Data range** | | 1.5 Å – 10 Å | | 1.5 Å – 10 Å |
| **Data summation number** | | 1 frame (5 s) | | 1 frame (5 s) |
| **Number of refined parameters** | | 10 | | 7 |
| **Scale** | | 0.49 | | 0.32 |
| **$R_w$** | | 0.27 | | 0.52 |
| **wA** | | 0.13 | | |
| **wasym** | | 15.0 | | |
| **wlam** | | 5.30 | | |
| **wphi** | | 0.76 | | |
| **wr0** | | 2.15 | | |
| **wsig** | | 0.53 | | |

**Table S13** Refined values for data collected after 13 min of reaction at 100 °C using $ReO_3$ as the structural starting model (Figure 8D).

| | **$ReO_3$** | |
|---|---|---|
| | **Initial values** | **100 °C, 13 min** |
| **Space group** | *I* m -3 | |
| **Lattice par., a=b=c (Å)** | 7.4456(2) | 7.5574(8) |
| **Number of refined parameters** | | 6 |
| **Data range** | | 1.5 Å – 20 Å |
| **$R_w$** | | 0.57 |
| **Scale factor** | | 0.32 |
| **$U_{iso}$ Nb ($Å^{-2}$)** | | 0.0089 |
| **$U_{iso}$ O ($Å^{-2}$)** | | 0.097 |
| **$\delta_2$ ($Å^2$)** | | 2.68 |
| **Sp-diameter (Å)** | | 16.09 |

**Table S14** Refined values for data collected after 13 min of reaction at 100 °C using $HNbO_3$ as the structural starting model (Figure S15).

| | **$HNbO_3$** | |
|---|---|---|
| | **Initial values** | **100 °C , 13 min** |
| **Space group** | *I* m -3 | |
| **Lattice par., a=b=c (Å)** | 7.645(2) | 7.56 |
| **Cell vol., V ($Å^3$)** | 446.82 | |
| **Number of refined parameters** | | 6 |
| **Data range** | | 1.5 Å – 20 Å |
| **$R_w$** | | 0.48 |
| **Scale factor** | | 0.32 |
| **$U_{iso}$ Nb ($Å^{-2}$)** | | 0.0094 |
| **$U_{iso}$ O ($Å^{-2}$)** | | 0.041 |
| **$\delta_2$ ($Å^2$)** | | 1.20 |
| **Sp-diameter (Å)** | | 16.50 |

**Table S15** Refined values for data collected after 13 min of reaction at 100 °C using H-$Nb_2O_5$ as the structural starting model (Figure S16 A-C).

| | **H-$Nb_2O_5$** | | | |
|---|---|---|---|---|
| | **Initial values** | **100 °C , 13 min (C)** | **100 °C , 13 min (C)** | **100 °C , 13 min (D)** |
| **Space group** | *P* 1 2/m 1 | | | |
| **Lattice par., a (Å)** | 21.153(7) | 20.74 | 20.64 | 20.58 |
| **Lattice par., b (Å)** | 3.8233(5) | 3.77 | 3.78 | 3.79 |
| **Lattice par., c (Å)** | 19.3560(50) | 20.10 | 20.029 | 19.90 |
| **Lattice par., β (°)** | 119.80(2) | 120.94 | 120.44 | 119.99 |
| **Cell vol., V ($Å^3$)** | 1358.4 | | | |
| **Number of refined parameters** | | 8 | 8 | 35 |
| **Data range** | | 1.5 Å – 20 Å | 3.4 Å – 20 Å | 3.4 Å – 20 Å |
| **$R_w$** | | 0.61 | 0.60 | 0.39 |
| **Scale factor** | | 0.38 | 0.50 | 0.54 |
| **$U_{iso}$ Nb ($Å^{-2}$)** | | 0.0009 | 0.0024 | 0.0017 |
| **$U_{iso}$ O ($Å^{-2}$)** | | 0.0046 | 0.0029 | 0.0033 |
| **Sp-diameter (Å)** | | 17.56 | 16.070 | 18.84 |

| **Atomic positions for H-$Nb_2O_5$** | | | | | | |
|---|---|---|---|---|---|---|
| | **Initial values** | | | **Refined values** | | |
| **Label** | x (Å) | y (Å) | z (Å) | x (Å) | y (Å) | z (Å) |
| **Nb1** | 0 | 0.2285(3) | 0 | 0.000000 | 0.179043 | 0.000000 |
| **Nb2** | 0.5 | 0 | 0.5 | 0.500000 | 0.000000 | 0.500000 |
| **Nb3** | 0.16430(3) | 0 | 0.00353(3) | 0.165001 | 0.000000 | 0.017419 |
| **Nb4** | 0.23623(3) | 0 | 0.23134(3) | 0.248426 | 0.000000 | 0.232997 |
| **Nb5** | 0.30284(3) | 0 | 0.45456(3) | 0.292427 | 0.000000 | 0.483580 |
| **Nb6** | 0.36087(3) | 0 | 0.04553(3) | 0.357421 | 0.000000 | 0.050262 |
| **Nb7** | 0.43325(3) | 0 | 0.27741(4) | 0.451415 | 0.000000 | 0.271137 |
| **Nb8** | 0.56270(3) | 0 | 0.09346(3) | 0.532910 | 0.000000 | 0.092918 |
| **Nb9** | 0.62943(3) | 0 | 0.32219(3) | 0.636956 | 0.000000 | 0.307538 |
| **Nb10** | 0.09353(3) | 0.5 | 0.20187(3) | 0.105737 | 0.500000 | 0.210789 |
| **Nb11** | 0.15921(3) | 0.5 | 0.42410(3) | 0.168956 | 0.500000 | 0.440706 |
| **Nb12** | 0.70330(3) | 0.5 | 0.12414(3) | 0.705899 | 0.500000 | 0.141844 |
| **Nb13** | 0.77056(3) | 0.5 | 0.35151(3) | 0.770681 | 0.500000 | 0.349591 |
| **Nb14** | 0.89895(3) | 0.5 | 0.16353(3) | 0.905035 | 0.500000 | 0.181087 |
| **Nb15** | 0.96461(3) | 0.5 | 0.38928(4) | 0.961990 | 0.500000 | 0.390516 |

## *Ex situ experiment: Synthesis of niobium oxide nanoparticles ambient pressure and 100 °C*

A synthesis similar to the *in situ* experiment was done in our home laboratory to produce particles for TEM and SAXS characterization, as well as *ex situ* PDF analysis.
Total scattering data of the as-prepared solution was measured for 50 hours on a Panalytical Empyrean Series 3 with an Ag-source (wavelength of 0.56 Å), equipped with a Galipix detector. Background scattering was collected in the same way with a capillary filled with pure benzyl alcohol. The total scattering data was Fourier Transformed to obtain the PDF using PDFgetX3.[4] The following parameters were used for the data reduction: : $Q_{min}$=1.3 Å$^{-1}$, $Q_{max}$=13.5 Å$^{-1}$, $Q_{maxinst}$=18.4 Å$^{-1}$ and rpoly=0.9.
TEM images were collected on a Tecnai T20 G2 200 kV TEM at the National Center for Micro-and Nanofabrication at the Technical University of Denmark.
SAXS and WAXS data were measured on a SAXSLab instrument (JJ-X-ray, Denmark) at the Niels Bohr Institute, University of Copenhagen. The instrument is equipped with a 100XL + microfocus sealed X-ray tube from Rigaku that produces a photon beam with a wavelength of 1.54 Å and a 2D 300 K Pilatus detector from Dectris. The 2D scattering patterns were azimuthally averaged, normalised for sample transmission, primary beam intensity and exposure time and corrected for detector inhomogeneities using Saxsgui. Scattering patterns from an empty quartz capillary and a capillary with benzyl alcohol were measured as backgrounds. The background subtraction was done in a Python script. The scattering angles 2θ were converted to a Q-scale: $Q = \frac{4\pi}{\lambda}\sin\left(\frac{2\theta}{2}\right)$. Simulated SAXS formfactor models were calculated in Diffpy-CMI, which uses SASVIEW functions, with a monodisperse sphere model.

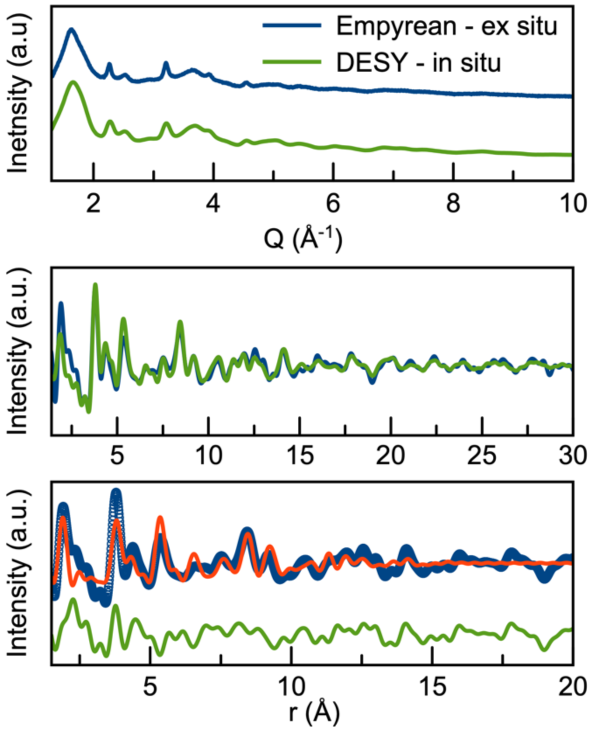


**Fig. S18** Top: Total scattering data collected after 13 min of reaction at 100 °C and ambient pressure at the Beamline P.01. DESY (in-situ) compared an experiment performed in our home laboratory. Middle: PDFs obtained for the two datasets. Bottom: Structural refinement on PDF collected for the ex situ experiment using $ReO_3$ as the structural starting model.

**Table S16** Refined values obtained for data collected on Panalytical Empyrean using $ReO_3$ as the structural starting model (Figure S17).

| | **$ReO_3$** | |
|---|---|---|
| | **Initial values** | **100 °C, 13 min *ex situ*** |
| **Space group** | *I* m -3 | |
| **Lattice par., a=b=c (Å)** | 7.4456(2) | 7.56 |
| **Number of refined parameters** | | 6 |
| **Data range** | | 1.5 Å – 20 Å |
| **$Q_{damp}$, $Q_{max}$, $Q_{min}$** | | 0.011, 1.3 Å, 13.5 Å |
| **$R_w$** | | 0.61 |
| **Scale factor** | | 0.019 |
| **$U_{iso}$ Nb (Å$^{-2}$)** | | 0.0096 |
| **$U_{iso}$ O (Å$^{-2}$)** | | 0.11 |
| **Sp-diameter (Å)** | | 17.21 |
| **$\delta_2$ (Å$^2$)** | | 3.46 |

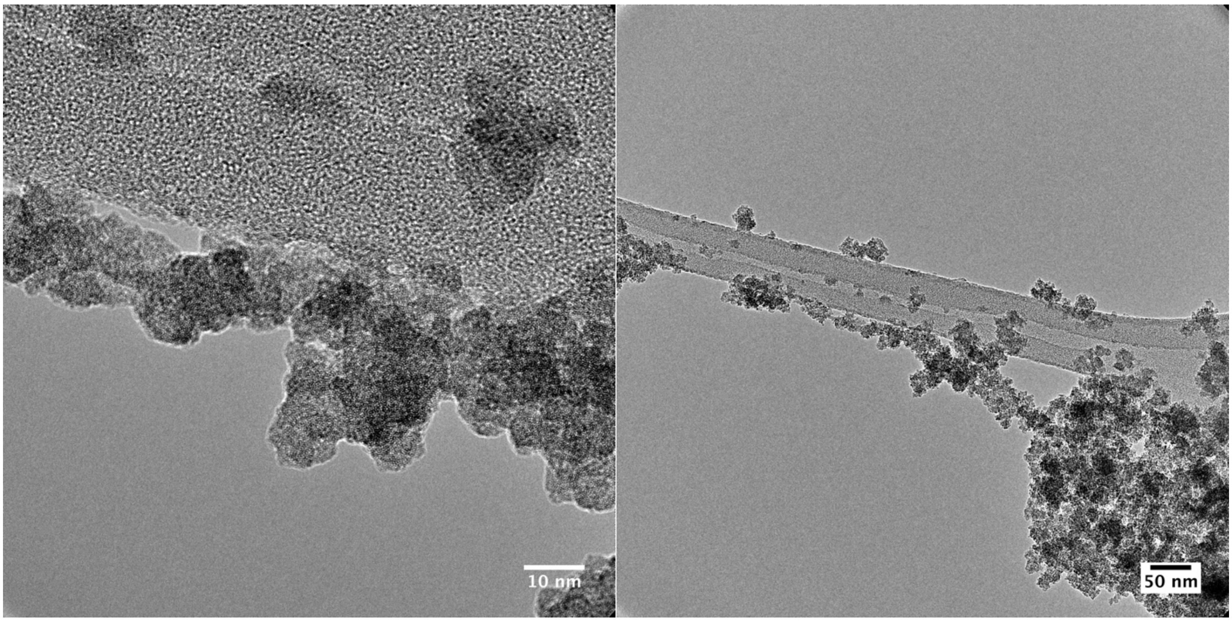


**Fig. S19** TEM of particles formed in the *ex situ* set-up at 100 °C.

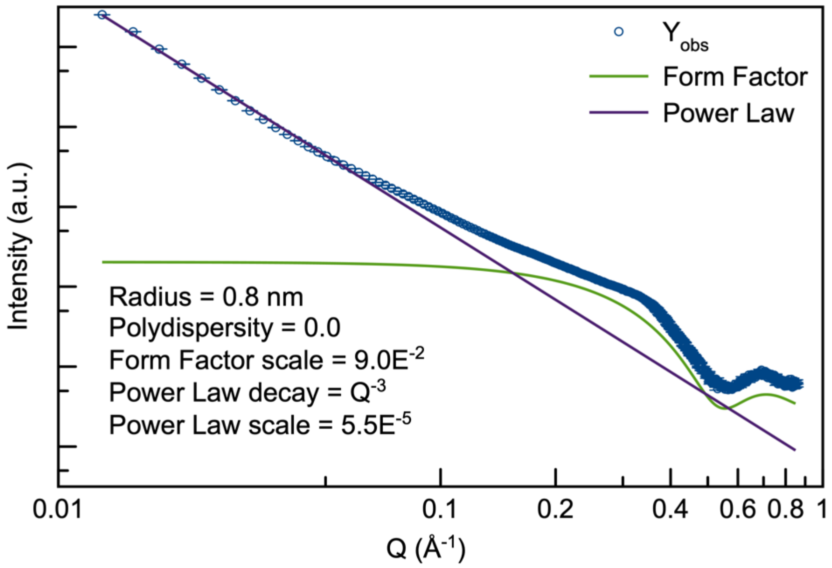


**Fig. S20** SAXS data obtained for the as-synthesized niobium oxide nanoparticles. A calculated SAXS form factor assuming a spherical shape and a particle radius of 0.8 nm gives a good description of the data in the high Q-regime of the data. A power law can describe the aggregation of particles into a two-dimensional network, seen in the low Q-regime.

## References


1. H. Schafer, R. Gruehn and F. Schulte, *Angew. Chem. Int. Edit.*, 1966, **5**, 40-52.
2. P. Juhas, C. L. Farrow, X. H. Yang, K. R. Knox and S. J. L. Billinge, *Acta Cryst.*, 2015, **71**, 562-568.
3. M. Zobel, R. B. Neder and S. A. J. Kimber, *Science*, 2015, **347**, 292-294.
4. P. Juhas, T. Davis, C. L. Farrow and S. J. L. Billinge, *J. Appl. Cryst.*, 2013, **46**, 560-566.
